# Statistical estimation theory detection limits for label-free imaging

**DOI:** 10.1117/1.JBO.29.S2.S22716

**Published:** 2024-09-05

**Authors:** Lang Wang, Maxine Varughese, Ali Pezeshki, Randy Bartels

**Affiliations:** a Morgridge Institute for Research, Madison, Wisconsin, United States; b Colorado State University, Fort Collins, Colorado, United States

**Keywords:** label-free microscopy, nonlinear microscopy, coherent Raman, quantitative phase, photothermal, transient absorption

## Abstract

**Significance:**

The emergence of label-free microscopy techniques has significantly improved our ability to precisely characterize biochemical targets, enabling non-invasive visualization of cellular organelles and tissue organization. However, understanding each label-free method with respect to the specific benefits, drawbacks, and varied sensitivities under measurement conditions across different types of specimens remains a challenge.

**Aim:**

We link all of these disparate label-free optical interactions together and compare the detection sensitivity within the framework of statistical estimation theory.

**Approach:**

To achieve this goal, we introduce a comprehensive unified framework for evaluating the bounds for signal detection with label-free microscopy methods, including second-harmonic generation, third-harmonic generation, coherent anti-Stokes Raman scattering, coherent Stokes Raman scattering, stimulated Raman loss, stimulated Raman gain, stimulated emission, impulsive stimulated Raman scattering, transient absorption, and photothermal effect. A general model for signal generation induced by optical scattering is developed.

**Results:**

Based on this model, the information obtained is quantitatively analyzed using Fisher information, and the fundamental constraints on estimation precision are evaluated through the Cramér–Rao lower bound, offering guidance for optimal experimental design and interpretation.

**Conclusions:**

We provide valuable insights for researchers seeking to leverage label-free techniques for non-invasive imaging applications for biomedical research and clinical practice.

## 1 Introduction

Optical imaging provides a method of observing biological systems that is particularly powerful for studying dynamics in live specimens. Information obtained from optical microscopes is derived from the light collected from the specimen. In one widespread approach, exogenous labels (often molecular dyes or fluorophores) are applied to interrogate the behavior of cells and tissues, such as nucleic acids, cytoplasm, extracellular proteins, or particular biomolecules. Despite the incredible power that comes from the specificity of the application of these labels, the labels carry their own problems. Many are toxic or severely disrupt biological function, complicating the interpretation of data from imaging experiments. In addition, the introduction of external labels is often impeded through physical processes, such as the need for labels to diffuse through tissue or pass through the blood–brain barrier.

An alternative strategy for optical microscopy, label-free imaging, uses intrinsic optical properties for imaging biological samples. Such strategies provide a rich palette of light-molecule interactions that produce an optical signal from which an optical microscope image may be formed. In this special issue of the *Journal of Biomedical Optics* (JBO), we are celebrating the wide-ranging contributions that our dear colleague Gabi Popescu made to this field. Gabi was a big champion and cheerleader in this field, and his enthusiasm for the widespread utility of label-free imaging was infectious.

There is a wide range of label-free imaging modalities. Each modality probes particular features of the specimen, and each exhibits a sensitivity that depends on the sample properties and the experimental scenario. However, the field lacks a comprehensive comparison among various techniques to determine when each method will provide useful information, as well as an assessment of the detection sensitivity of these methods. In this work, we develop a general model for label-free signal generation to facilitate the investigation of the relative performance of these label-free imaging methods.

Our analysis considers a universal light–matter interaction mechanism for label-free imaging techniques, and then, we apply the tools of statistical information theory to study the detection limits with label-free imaging methods. This strategy establishes bounds on the detection sensitivity of label-free microscopy. Note that we do not treat label-free methods based on the autofluorescent properties of a small set of endogenous biomolecules as these methods cannot be incorporated into our general optical signal model.

A wide range of label-free optical interactions have been exploited for optical microscopy. These optical modalities universally rely on optical spectroscopy of illumination light and the methods in which the light–matter interactions in the specimen modify light propagation, polarization, or color. Label-free imaging often relies on linear optical scattering, in which spatial variations in the optical susceptibility, δε, distort light propagation through a specimen. To recover the three-dimensional variation in optical susceptibility, a range of optical methods can record quantitative changes in the optical phase and amplitude and solve an inverse scattering problem. Although such quantitative phase microscopy methods^1^^–^^3^ can be ubiquitously applied to specimens, optical spectroscopy shows little dispersion, and as a result, it has difficulty differentiating among particular molecular species.^4^ Nonlinear optical scattering processes of second- and third-harmonic generations (SHG and THG) can occur for a large incident optical field strength. These nonlinear scattering mechanisms convert incident light into a new color and reveal tissues formed from organized distributions of structural proteins (SHG)^5^^–^^12^ or morphologies such as cell membranes and small lipid bodies (THG).^13^^–^^19^

The rise of label-free microscopy has facilitated our ability to chemically specify biochemical targets, allowing us to visualize cellular organelles without perturbing the biological dynamics. Because the identification and observation of the behavior of biomolecules provide critical insight into biological systems, methods that can provide label-free biochemical detection are highly sought after and form the basis of several label-free imaging methods that differentiate molecules based on their vibrational spectral fingerprints^20^^–^^24^ or based on the excited state decay dynamics.^25^^–^^28^ The simplest vibrational spectral measurements exploit direct mid-infrared absorption at vibrational frequencies for which motion induces a change in the molecular dipole, thus producing direct optical absorption with incident light that matches the vibrational energy.^29^^–^^32^ Alternatively, the Raman-active vibrational spectroscopy can be probed; the vibrational motion leads to a change in molecular polarizability and thus drives inelastic optical scattering, in which scattered light either gains or loses a quanta of vibrational energy.^33^^,^^34^ Conventional Raman spectroscopy and imaging are limited in detection sensitivity because they rely on spontaneous Raman scattering, a rare process. Stimulated Raman scattering techniques, such as coherent anti-Stokes Raman scattering (CARS),^35^^–^^38^ coherent Stokes Raman scattering (CSRS),^39^ stimulated Raman scattering (SRS),^40^^,^^41^ or impulsive stimulated Raman scattering (ISRS),^42^^–^^49^ greatly increase the Raman signal scattering.

An advantage of stimulated spectroscopic interactions for the imaging of molecular targets is that the rate of signal generation may be elevated relative to the naturally excited state relaxation times that constrain the fluorescent imaging rates. The rate of signal generation can be increased in pump–probe experimental arrangements such as transient absorption (TA), excited state absorption (ESA), stimulated emission (SE), or ground state depletion (GSD)^25^^,^^27^^,^^50^^,^^51^ imaging methods. In this family of interactions, a pump pulse drives electronic absorption that perturbs the transmitted power of a time-delayed probe pulse.^52^

Following the excitation of a molecular chromophore, electrons promoted to an excited state will relax back down to the ground state, and this excess energy is thermalized. Thermalization of the deposited energy heats the region surrounding the chromophore—producing an increase in temperature and pressure. These two perturbations are exploited for photoacoustic (PA)^53^ and photothermal (PT)^54^^,^^55^ detection mechanisms. We study the latter here because the detection is optical and thus relevant to our signal model. PT interactions can be driven by optical absorption^56^ or vibrational state transitions^57^^–^^59^ that leave residual energy in the molecule. The temperature change induced by the energy dissipation following optical excitation produces a small change in the effective linear optical susceptibility, δε. This differential change δε can then be extracted by comparing optical phase images or changes in optical scattering following excitation to those taken at thermal equilibrium.

To link all of these disparate label-free optical interactions together, we consider a description that can incorporate the signal model for each of these modalities. The signal model that we develop links all label-free imaging methods together to highlight the key underlying signal generation mechanism. To assess their relative detection limits, we employ the general signal model and compute the information available in measurements using statistical estimation theory. This model allows for a direct comparison of the detection sensitivity among all methods. Specifically, we consider scattering-induced changes in an optical imaging field produced by a spherical perturbation of optical susceptibility δε. A model of the imaging field that has passed through an optical microscope is developed, so a model of the signal detection probability may be constructed. This signal model accounts for shot noise in optical detection, capturing the limiting case of optical detection in the standard quantum limit. On the basis of this model, the measurement information is quantified by Fisher information, and the fundamental limits in estimation precision for δε are assessed by the Cramér–Rao lower bound (CRLB). The effective susceptibility perturbation is then calculated for the label-free imaging methods presented in the introduction, so detection bounds of molecular concentration, or other parameters of interest, may be established with the general model. This general analysis can be applied to any label-free spectroscopy or imaging method, and we hope it will be a valuable tool for assessing label-free imaging experiments.

## 2 Imaging Model for Signal Detection

**Fig. 1:**
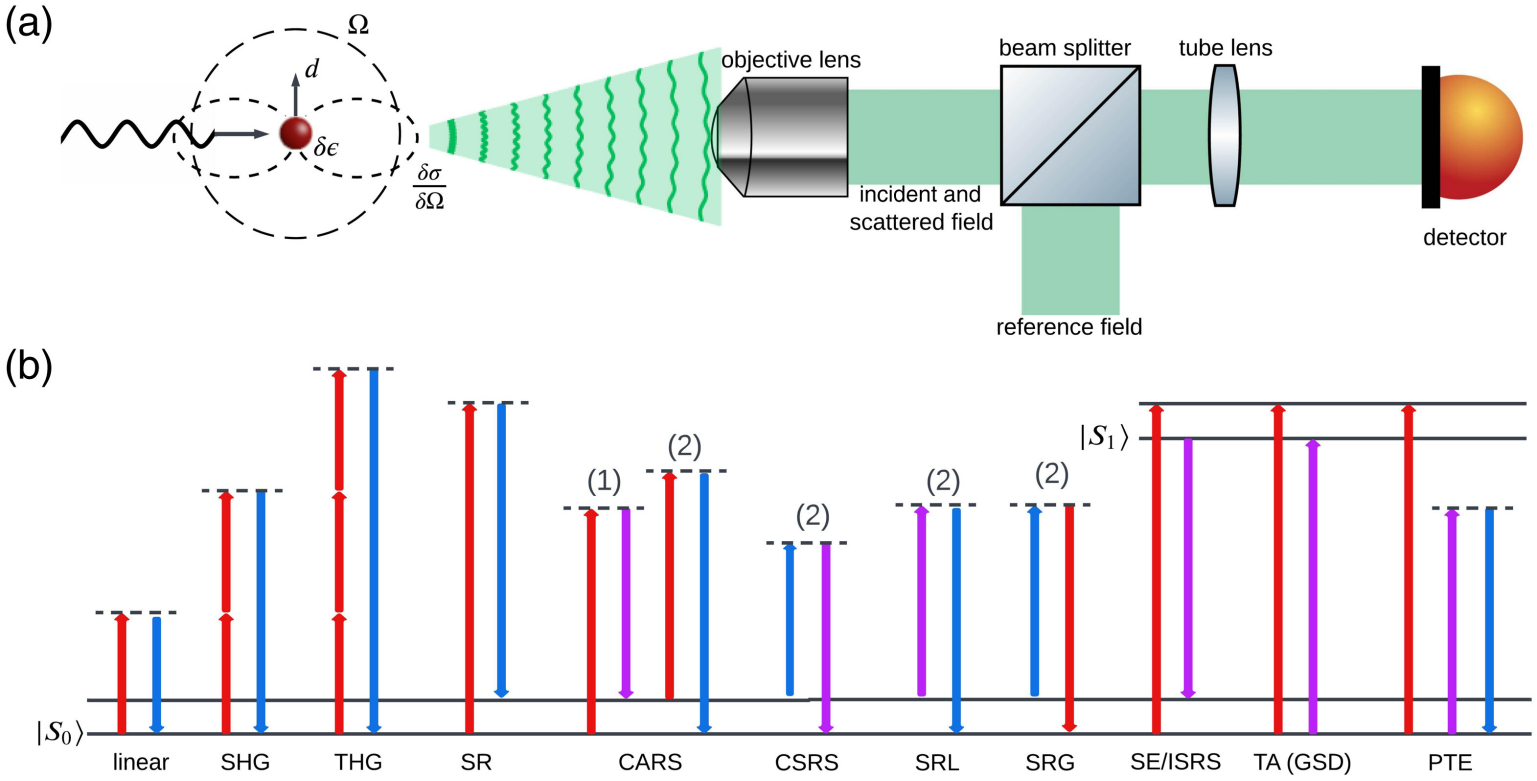
Model and label-free imaging techniques. (a) From left to right: scattering of the particle, 4-f imaging configuration, and measurement by a camera. In the scattering event, the particle is illuminated by an incident light, forming a dipole moment $\overset{¯}{\mu }$. Scattering property of the dipole in the far-field region. (b) Contrast mechanisms of the imaging methods. Methods are classified into dark-field and bright-field methods. In the pump–probe methods, the red arrow denotes the pump, the purple arrow denotes the probe, and the blue arrow denotes the scattering, which is the spatial frequency of the particle to be imaged. Notice that CARS, CSRS, SRL, and SRG share the same step (1), so only step (2) is shown for CSRS, SRL, and SRG.

Our universal model for label-free signal generation is based on the optical system illustrated in Fig. 1. We consider an object that consists of a spherical perturbation of optical susceptibility, $\delta \varepsilon =\varepsilon_{s}-\varepsilon_{b}$, a change in the relative dielectric permittivity of the sphere, $\varepsilon_{s}$, relative to a background relative dielectric permittivity, $\varepsilon_{b}$. The sphere has a radius a≪λ that is smaller than the incident field wavelength λ. The signal model is determined by the light that is imaged from the object space to the image space through a 4-f optical microscope; we closely follow the theoretical analysis of the image of a dipole in an optical microscope.^60^ Once the model for the signal is obtained, we apply the tools of statistical estimation theory to establish the bounds on the precision with which we estimate δε.

### 2.1 Dipole Moment of a Sub-wavelength Particle

Our particle lies in an object space, with coordinates r=(x,y,z) centered at the origin $r_{0}=(0,0,0)$, and is illuminated by an incident optical field Ei(r,t)=E0ur(r)ut(t)exp(inbkjz0)ϵ^. We assume that the incident beam propagates along z, so the beam polarization vector, ϵ^, lies in the transverse plane with a surface normal aligned with z. The incident beam has a peak electric field strength of $E_{0}$, with a spatial variation of field amplitude that varies due to beam diffraction described by $u_{r}(r)$. This diffracted field is normalized such that $|u_{r}|\leq 1$. For cases of a pulse, we assume that $u_{t}$ is a complex temporal envelope normalized to $|u_{t}|\leq 1$ and that the pulse is a member of a pulse train with a repetition rate of $\nu_{r}$. Note that, for an unpulsed continuous wave beam, $u_{t}=1$. We assume that the illumination beam is centered at the origin such that $u_{r}(r_{0})=1$. The free space wavenumber, $k_{j}=2\pi /\lambda_{j}$, is increased by the object space background refractive index, $n_{b}=\sqrt{\varepsilon_{b}}$, leading to $k=n_{b}\;k_{j}$. The beam transverse intensity profile is $I(r_{⊥},t)=I_{0}|u_{r}(r_{⊥},z){|}^{2}|u_{t}(t){|}^{2}$ at an axial plane z, where the transverse spatial coordinate vector is $r_{⊥}=(x,y)$. Definitions of the beam area, $A_{b}=I_{0}^{-1}\int I(r_{⊥},0)d^{2}r_{⊥}$, and the pulse temporal duration, $\tau_{p}=I_{0}^{-1}\int I(0,t)dt$, make use of this transverse intensity profile. Finally, we note that the average power of the incident beam is $p_{i}=\nu_{r}\int I_{i}(r_{⊥},t)dt\;d^{2}r_{⊥}=\nu_{r}\tau_{p}A_{b}I_{0}\equiv A_{b}I_{a}$, which can be written in a very simple form using the average intensity $I_{a}=\nu_{r}\tau_{p}I_{0}$.

Due to constraints of optical diffraction, the beam area is on the order of $\lambda^{2}$ or larger. Consequently, scattering by the sub-wavelength centered on the illumination beam is produced by an oscillating electric dipole moment driven by the field at the peak of the incident beam, $\mu =\tilde{\alpha }E_{i}(r_{0})$, where we assume that the particle is centered on the beam. The parameter $\overset{˜}{\alpha }\equiv \varepsilon_{0}\alpha$ is a complex quantity describing the propensity of a scatterer to produce a polarization in response to an applied electric field. We separate this polarizability into a product of the dielectric permittivity of free space, $\varepsilon_{0}$, and the complex-valued polarizability volume α. Even for a real-valued optical susceptibility perturbation, δε, the polarizability will be complex due to rescattering described by the radiative reaction term.^61^ However, because we consider $k_{j}a\ll 1$ and δε≪1, we may reliably approximate the polarizability volume by α≈Vδε, where the volume of the sphere is $V=(4\pi /3)a^{3}$.

Within the approximations presented here, the oscillating dipole moment, μ, radiates a dipole electric field. This radiation is the universal physical origin of our label-free signal used for probing a specimen. The frequency at which the induced dipole oscillates is determined by the particular label-free interaction, and the interaction can either be an elastic scattering process, in which the wavelength stays the same as the incident light, or an inelastic scattering process, in which there is a change in the optical wavelength. In addition, the effective susceptibility perturbation will be determined by the number of molecules contributing within the perturbation volume, V, and the coherence of the interaction that drives the susceptibility perturbation. The specific cases for label-free imaging are discussed later in this article.

### 2.2 Dipole Scattering and Cross-sections

To appreciate signal levels, we consider the expression for the extinction, scattering, and absorption cross-sections of the label-free perturbation.^62^ The extinction cross-section reads as σext=(kj/nb)V Im{δε}, whereas the scattering cross-section is given by $\sigma_{s}=(k_{j}^{4}V^{2}/6\pi )|\delta \varepsilon {|}^{2}$. The difference is the absorption cross-section, $\sigma_{\mathrm{abs}}=\sigma_{\mathrm{ext}}-\sigma_{s}$. Note that these cross-section values will be composed of molecular components within our scattering sphere and the contribution of the effective polarizability of the set of molecular components to the total δε. As a consequence, the total cross-section values depend on the coherence among induced dipole moments between each molecule within the sphere. Each scenario is treated separately later in the article.

With the cross-section for the sub-wavelength sphere, we can estimate the extinguished and scattered average powers as $p_{\sigma }=\nu_{r}\sigma \int I_{i}(0,t)dt$. Making use of the expression for the incident average power, we write $p_{\sigma }=(\sigma /A_{b})p_{i}$, scaling as the ratio of the cross-section and the incident beam area. For a fixed incident optical power budget, it is desirable to focus the incident beam tightly to minimize $A_{b}$ and thus maximize the intensity of the illumination beam. In a high numerical aperture (NA) focusing limit, an incident beam focused on the origin can be approximated with a three-dimensional Gaussian distribution $u_{r}(r)=\exp(-\rho^{2}/w_{0}^{2})\exp(-z^{2}/w_{z}^{2})$. Here, $\rho =\sqrt{x^{2}+y^{2}}$ is the radial transverse coordinate, and the radii of the focused beam are $w_{0}=0.52n_{b}\lambda /\mathrm{NA}$ and $w_{z}=0.76\lambda /[1-\sqrt{1-(\mathrm{NA}/n_{b}{)}^{2}}]$.^63^ This produces a beam area of $A_{b}=0.42(n_{b}/\mathrm{NA})\lambda^{2}$. Combining these expressions, we find that the power (scattered, extinguished, or absorbed) reads as $p_{\sigma }\approx 2.4(\mathrm{NA}/n_{b}{)}^{2}(\sigma /\lambda^{2})p_{i}$. The coefficient in front of the incident power is a small number, indicating that much of the incident power is unperturbed. This sets a level of background light that usually degrades sensitivity and must be accounted for in the signal model. For a complete model, we compute the image of the excitation beam and scattered beam through a 4-f imaging system.

### 2.3 Signal Obtained from the Image of the Dipole Through a Microscope

To compute the signal produced from a field imaged from the object region into an image region, we follow the derivation of the image of a dipole field produced with a high NA imaging system.^60^ The object field may be expanded into a set of transverse spatial frequencies, $k_{⊥}=(k_{x},k_{y})$, at a reference plane, here z=0, providing the expression of the object field

$$E_{\mathrm{obj}}(r)=\int e(k_{⊥})e^{i(k_{⊥}\cdot r_{⊥}+\gamma_{o}z)}d^{2}k_{⊥}.$$ (1)

The transverse spatial frequency spectral amplitude is denoted by $e(k_{⊥})$, and the angular spectral propagator can be used to express the field at a plane other than z=0 with a transverse spatial frequency phase determined by the axial spatial frequency for propagation, $\gamma_{0}=\sqrt{n_{b}^{2}k_{j}^{2}-{\left\|k_{⊥}\right\|}^{2}}$ of the wave vector, at $k=(k_{⊥},\gamma_{o})$, for each transverse spatial frequency.

The object field is mapped to an image space where we place a detector using a 4-f optical imaging system. As indicated in Fig. 1, the focal lengths of the imaging system are $f_{1}$ and $f_{2}$, leading to a magnification of the object field by the factor $M=-f_{2}/f_{1}$. When mapping the object field to the image space in air, the wave vector is transformed such that $k^{\prime }=(k_{{⊥}^{\prime }},\gamma_{i})$, where $k_{⊥}^{\prime }=(k_{x}/M,k_{y}/M)$ and $\gamma_{i}=\sqrt{k_{j}^{2}-{\left\|k_{⊥}^{\prime }\right\|}^{2}}$. The image of the object field, labeled by the image–space coordinates $r_{1}=(r_{1⊥},z_{1})$, at the in-focus plane of $z_{1}=0$ may be expressed in terms of the object space transverse spatial frequencies as

$$E_{\mathrm{img}}(r_{1})=M\int \sqrt{\frac{\gamma_{o}}{\gamma_{i}}}H(Mk_{⊥}^{\prime })e_{1}(Mk_{⊥}^{\prime })e^{ik_{⊥}^{\prime }\cdot r_{1⊥}}d^{2}k_{⊥}^{\prime }.$$ (2)

Moreover, the polarization of the dipole field may be decomposed into $\hat{s}$ and $\hat{p}$ polarization directions. The imaging system transforms the object field polarization through a unitary rotation, thus preserving the magnitude of the transverse spatial frequency components, so $\left|e_{1}|=|e\right|$. The coherent transfer function of the 4-f imaging system, $H(k_{⊥})$, is a low-pass transverse spatial frequency filter with a cutoff spatial frequency, $k_{c}=2\pi \mathrm{NA}/\lambda_{j}$, determined by the NA of the imaging objective lens ($f_{1}$).

We assume that the NA of the imaging objective lens does not restrict the collection of the illumination beam, which implies that the beam is simply expanded by the magnification factor with a corresponding drop in field amplitude such that the full power of the incident excitation beam is transmitted through the imaging system. The label-free signal originates from the source, $Q=(k_{0}^{2}/\varepsilon_{0})\mu$, that is produced by the induced dipole moment. The transverse spatial frequency spectrum of the forward-propagating component of the dipole field reads as

eQ(k⊥)=i8π2γo[Q−(Q·k^)k^]. (3)

Here, k^=k/‖k‖, and we exploited the fact that we consider an object to be located at the origin of the coordinate system. The power of the field scattered in the forward direction is given by a prior work;^60^ the formula is $p_{\mathrm{sob}}=(2\pi^{2}/\mu_{0}\omega )\int |e(k_{⊥}){|}^{2}\gamma_{o}d^{2}k_{⊥}$. Applying this to the dipole scattered field, we find that $p_{\mathrm{sob}}=\sigma_{s}I_{0}/2$, which is half of the total scattered power. The other half of the power propagates in the backward direction. Due to the finite NA of the imaging objective lens, the power of the image of the dipole emission is reduced to $p_{\text{sim}}=\eta_{c}p_{\mathrm{sob}}$, where the efficiency of the forward-scattered power by the object is given by $\eta_{c}=1-\sqrt{1-{\mathrm{NA}}^{2}}+({\mathrm{NA}}^{2}/4)\sqrt{1-{\mathrm{NA}}^{2}}$.

To admit a wide range of experimental arrangements, we consider the following total field in the image space:

$$E_{\mathrm{tot},\mathrm{im}}=aE_{\mathrm{ex},\mathrm{im}}+E_{Q,\mathrm{im}}+E_{r}\equiv \overset{˜}{r}E_{\mathrm{ex},\mathrm{im}}+E_{Q,\mathrm{im}}.$$ (4)

This total field consists of the dipole field image, $E_{Q,\mathrm{im}}$; the image of the excitation field, $E_{\mathrm{ex},\mathrm{im}}$; and a reference field, $E_{r}=rE_{\mathrm{ex},\mathrm{im}}$. We assumed that the reference field is a replica of the image of the illumination field, scaled by a complex factor r=R exp(iϕ), where R is the relative amplitude and ϕ is the relative phase of the reference beam. In addition, we multiply a factor a by the image of the excitation field, so we can set a=1 to represent bright-field imaging and a=0 for the case of dark-field imaging, in which the unscattered field is not collected. We define a generalized complex reference amplitude as $\overset{˜}{r}=a+r$.

In our Fisher information analysis, we consider both cases in which a single detector collects some fraction of the total signal power or we send the image onto a camera to capture the signal. In both cases, the information content is the same, and the measurement on each pixel is uncorrelated. As a result, the relevant parameter from the signal model starts with the total power of the signal, which is computed from the total transverse spatial frequency amplitude. This total power is the sum of three terms, $p_{t}=|\overset{˜}{r}{|}^{2}p_{i}+p_{\mathrm{sig}}+p_{\mathrm{int}}$. The first two power terms have already been computed, and the interference power term, pint=(4π2r˜*/μ0ω)∫|H(k⊥)|2 Re{eQ(k⊥)·eex*(k⊥)}γod2k⊥, arises from mixing between the dipole source transverse spatial frequency distribution given in Eq. (3) and the excitation beam transverse spatial frequency distribution, $e_{\mathrm{ex}}(k_{⊥})=(4\pi^{2}{)}^{-1}\int u(r_{⊥})\exp(-ik_{⊥}\cdot r_{⊥})d^{2}r_{⊥}$. Assuming that we have a symmetric, unaberrated beam propagating along the optical axis that was produced by a uniformly filled illumination optic numerical aperture (${\mathrm{NA}}_{i}\leq \mathrm{NA}$), we find that pint=−I0kjV Im{r˜*δε}fNA, where $f_{\mathrm{NA}}=1-({\mathrm{NA}}_{i}/2n_{b}{)}^{2}$. Note that plane wave illumination is the limiting case in which ${\mathrm{NA}}_{i}\to 0$. The signal collected in a time interval Δt, for a photon energy $E_{\mathrm{ph}}=hc/\lambda_{j}$ and a detector quantum efficiency $\eta_{d}$ that has a surface area larger than the beam size, gives us a mean-detected photon count given by $N=\eta_{d}\Delta tp_{t}/E_{\mathrm{ph}}$

N=Ni(|r˜|2+12ηcσeff(j)Ab−kjVAb Im{r˜*δεeff(j)}ℓ(j)fNA)≡NiFN, (5)

where we define $N_{i}=\eta_{d}p_{i}/E_{\mathrm{ph}}$ as the mean number of detectable photons in the illumination beam and the single-photon signal function $F_{N}$. In addition, we define $\delta \varepsilon_{\mathrm{eff}}^{\left(j\right)}=NB^{\left(j\right)}\alpha^{\left(j\right)}$ for use with coherent nonlinear scattering as the effective susceptibility becomes dependent on the incident fundamental beam power and, of course, with CARS and CSRS, will depend on Stokes and pump beam powers. The parameters $B^{\left(j\right)}$ and $\ell^{\left(j\right)}$ are defined when coherent nonlinear scattering is discussed and account for pulse averaging effects and for the linear case, where j=1, $B^{\left(1\right)}=1$, and $\ell^{\left(1\right)}=1$. In addition, $\sigma_{\mathrm{eff}}=k_{j}^{4}V^{2}{\left|\delta \varepsilon_{\mathrm{eff}}\right|}^{2}/6\pi$ defines the effective scattering cross-section. The number density, N, of the molecules in the sphere leads to a total number of molecules of NV, each with a single-molecule polarizability (for j=1, where $\alpha^{\left(1\right)}$ is the molecule polarizability), and for j>1, we represent hyperpolarizabilities used to describe nonlinear scattering processes.

Due to the large parameter space of this analysis, we further simplify our notation for a utility that previews what will be useful in the Fisher information analysis that follows. In this simplified analysis, we separate the normalized flux, $F_{N}=|\overset{˜}{r}{|}^{2}+F_{N}^{\mathrm{df}}+F_{N}^{\mathrm{int}}$, into the reference, $|\tilde{r}{|}^{2}$; dark-field, $F_{N}^{\mathrm{df}}$; and the interference term of $F_{N}^{\mathrm{int}}$. To facilitate simple analysis with the Fisher information, the dark-field and interference terms are written as a constant that is proportional to the effective susceptibility perturbation, giving us the normalized flux for the dark-field case as $F_{N}^{\mathrm{df}}=\Gamma |\delta \varepsilon_{\mathrm{eff}}^{\left(j\right)}{|}^{2}$ and for the interference term, FNint=−H Im{r˜*δεeff(j)}. A comparison with Eq. (5) shows that $\Gamma =(\eta_{c}/2)k_{j}^{4}V^{2}/6\pi A_{b}$ and $H=k_{j}V\ell^{\left(j\right)}f_{\mathrm{NA}}/A_{b}$.

### 2.4 Probabilistic Model for Susceptibility Perturbation Estimation and CRLB

Having established the measurement model in terms of photon count for the particle susceptibility in label-free imaging, we now delve into the quantitative assessment of each imaging system’s sensitivity of the measurement data or, equivalently, the amount of information that the measurement data carry about the particle susceptibility. The statistical tools used are the Fisher information, J, and the CRLB, both of which are instrumental in quantifying the fundamental limits in estimation precision.

When only photon detection noise is present, noise in optical detection can be modeled as a Poisson process, in which the likelihood function for detecting Y=y photons is expressed as $f(Y=y;\delta \varepsilon_{\mathrm{eff}})=N^{y}e^{-N}/y!$ and N is the mean photon count. Here, we analyze the estimation precision of δε in the model given in Eq. (5) for a Poisson noise model. It is beneficial to define normalized Fisher information $\tilde{J}$ by the number of photons from incident light $N_{i}$ because the Fisher information scales linearly with respect to the signal strength. The stronger the detected signal, which requires an increased illumination power, the larger the Fisher information. The normalized Fisher information $\tilde{J}$ signifies the amount of information carried by a single incident photon about the object susceptibility perturbation. The Fisher information for this estimate, $J=N_{i}\tilde{J}$, can be separated into a product of the incident mean photon count $N_{i}$ and the normalized single-photon Fisher information, $\tilde{J}$, that provides information on the sensitivity for an experimental arrangement on the detection of the parameter of interest. The Fisher information and the CRLB are inherently connected as the CRLB is inversely proportional to the Fisher information. Serving as a theoretical lower limit on the variance of any unbiased estimator when evaluated at the true parameter value, the CRLB for any unbiased estimation of the susceptibility is, therefore, given by $\sigma_{\mathrm{CRLB}}^{2}=J^{-1}=N_{i}^{-1}{\tilde{J}}^{-1}$. The limit to the precision with which a single parameter is simply $\sigma_{\mathrm{CRLB}}=1/\sqrt{J}$. Multiparameter estimation is more complex because the Fisher information becomes a matrix that must be inverted to obtain the CRLB values for estimation precision on its diagonal.

The normalized Fisher information, $\tilde{J}=s_{N}^{2}/F_{N}$, is the ratio of the square of the single photon Fisher score, $s_{N}$, to the single-photon signal flux $F_{N}$. The Fisher score, which is the derivative of the log-likelihood function for the measurement with respect to the parameter of interest, establishes the sensitivity of the measurement with respect to the parameter of interest and helps quantify the amount of information that a set of data provides about the parameter of interest in a statistical model. In general, δε is a complex-valued parameter, so we consider the limiting cases in which our parameter of interest, δε, is either purely real, so $\delta \varepsilon \to \delta \varepsilon_{\mathrm{re}}$ or is purely imaginary, so $\delta \varepsilon \to i\delta \varepsilon_{\mathrm{im}}$. Then, we consider two cases: the normalized Fisher information for a real-valued susceptibility perturbation is given by ${\tilde{J}}_{\mathrm{re}}=(s_{N}^{\left(\mathrm{re}\right)}{)}^{2}/F_{N}^{\left(\mathrm{re}\right)}$, and the normalized Fisher information for an imaginary susceptibility perturbation is given by ${\tilde{J}}_{\mathrm{im}}=(s_{N}^{\left(\mathrm{im}\right)}{)}^{2}/F_{N}^{\left(\mathrm{im}\right)}$. For the real-valued case, we use

FN(re)=|r˜|2+Γ(δεeff(re))2−H Im{r˜*}δεeff(re) (6)

and

sN(re)=2Γδεeff(re)−H Im{r˜*}. (7)

Similarly, for the imaginary-valued case, we use

FN(im)=|r˜|2+Γ(δεeff(im))2−H Re{r˜*}δεeff(im) (8)

and

sN(im)=2Γδεeff(im)−H Re{r˜*}. (9)

The normalized Fisher information will be explored for a variety of label-free signal detection modalities and experimental arrangements. Recall that the experimental arrangement here is general, meaning that we can choose a dark-field scenario by setting a,R=0 all the way to bright-field interference, where a=1,R≠0. To account for these cases, we make use of the explicit expressions $|\overset{˜}{r}{|}^{2}=[a+R\;\cos(\phi ){]}^{2}+{\left[R\;\sin(\phi )\right]}^{2}$, Re{r˜*}=a+R cos(ϕ), and Im{r˜*}=−R sin(ϕ).

The Fisher information and the CRLB can be connected to the notion of signal and noise and thus the signal-to-noise ratio (SNR) that are commonly used to describe optics experiments. The change in the expected mean signal for the true parameter of interest value $\delta \epsilon_{\mathrm{eff},0}$ to be measured is approximated by $\Delta N_{s}\approx N_{i}s_{N}\Delta \delta \epsilon_{\mathrm{eff}}$ given that $\Delta \delta \epsilon_{\mathrm{eff}}$ is sufficiently small. Equivalently, when $\delta \epsilon_{0}$ is sufficiently small, the expected mean signal is given by $N_{s}\approx N_{i}s_{N}\delta \epsilon_{\mathrm{eff},0}$. The root mean square noise for the Poisson noise model here gives a noise of $N_{n}=\sqrt{N_{i}F_{N}}$. From these quantities, we may construct the SNR as $\mathrm{SNR}\equiv N_{s}/N_{n}$. This leads to the understanding of how the Fisher information and the CRLB relate to the balance between the signal and the noise. If a small change in the parameter of interest results in a larger change in the signal or the noise is lower, the Fisher information increases, and the CRLB decreases. Comparing this definition, we see that we may write $\mathrm{SNR}=\delta \varepsilon_{0}J^{1/2}=\delta \varepsilon_{0}/\sigma_{\mathrm{CRLB}}$. This suggests that the SNR can be comprehensively represented by the behavior of the maximum likelihood estimator (MLE) as it follows a Gaussian distribution with its mean equal to the true parameter value, and its variance approaches the CRLB when the number of measurements approaches infinity. Essentially, the variability in the MLE relative to the mean from different realizations of the same process is contingent on the level of noise power. A lower noise power results in diminished relative variability, highlighting the importance of noise control. Moreover, we may interpret the limit of detection as the object susceptibility perturbation at which SNR = 1 in our measurement, and therefore, $(\delta \varepsilon_{\mathrm{eff},0}{)}_{\min}=\sigma_{\mathrm{CRLB}}$.

In summary, higher Fisher information implies a lower CRLB, indicating that precise estimation of the parameter is attainable. Therefore, our following sensitivity analysis focuses on the calculation of the Fisher information for all label-free microscopy methods. Moreover, the Fisher information is broader than a calculation of the SNR, and significant differences can emerge between a simple SNR analysis and one based on Fisher information.^64^^,^^65^ Although SNR analysis is applied to a measure of total signal and total noise,^7^ it is not straightforward to apply such analysis to a multipixel detector such as a camera.^66^ The Fisher information analysis presented here is directly applicable to detection schemes in which a fraction of signal power is collected on a single photodetector or a camera is used and many pixels are used to collect the data over an image field of view. At first glance, these may not seem compatible; however, each pixel measurement is an independent event, and thus, the log-likelihood functions for each pixel add together, meaning that Fisher information will add for each pixel and becomes equivalent to the integrals used in our analysis. Thus, our results are equally valid for camera-based detection.^67^

## 3 Effective Susceptibility of Label-Free Optical Interactions

The normalized Fisher information, $\tilde{J}$, provides the contribution to the standard deviation of susceptibility perturbation estimation precision, ${\tilde{J}}^{-1/2}$, which we have seen also corresponds to the minimum detectable perturbation for a general scattering model. Each label-free interaction produces a susceptibility perturbation that is related to the intensity of the light that drives the light–matter interaction and sets bounds on the limit on how small of a concentration of the molecules under study may be detected. The coherence of the scattered light relative to the incident light also plays a role in the effective $\delta \varepsilon_{\mathrm{eff}}$ and the detection limits. In all cases, we consider a set of molecules contained within the scattering sphere volume, V, that produce a total detected light power. A summary of the results from this section is provided in Table 1. The effective susceptibility is grouped into several terms: $\delta \varepsilon_{\mathrm{eff}}^{\left(j\right)}=B^{\left(j\right)}N\alpha^{\left(j\right)}$. The number density, N, is set by the concentration of individual molecules in which each has an instantaneous effective polarizability, $\alpha^{\left(j\right)}$. For a linear interaction, this is the usual polarizability for linear scattering in the volume units as described earlier. For nonlinear interactions, these generalize to hyperpolarizabilities and then become intensity-dependent. The $B^{\left(j\right)}$ is an enhancement term that is obtained by computing a time-averaged signal from pulsed excitation and accounts for the nonlinear dependence of the effective susceptibility-based temporal averaging of the instantaneous nonlinear signal generation over the time course of a temporal pulse. The resulting effective linear susceptibility quantity can be used to compute the scattering and extinction cross-sections using the standard expressions for linear interactions, thus facilitating a comprehensive, global model.

**Table 1:** Summary of the effective susceptibility perturbation, $\delta \varepsilon_{\mathrm{eff}}$, for various label-free imaging modalities discussed in this paper, which are used to compute the scattered power and the term that dictates absorption and interference. This effective susceptibility is defined through the time-averaged label-free optical interaction strength that can be compared with each label-free imaging method. Through this averaging, the effective susceptibility is defined as $\delta \varepsilon_{\mathrm{eff}}=B^{\left(j\right)}N\alpha^{\left(j\right)}$. We assume that we have M=NV molecules within the interaction volume and that $\sigma_{a}^{\left(1\right)}$ denotes the single-molecule absorption coefficient.

	$\alpha^{\left(j\right)}$	$B^{\left(j\right)}$	$\delta \varepsilon_{\mathrm{eff}}$
SR	$\alpha^{\left(\mathrm{SR}\right)}=k_{s}^{-2}\sqrt{\frac{6\pi \sigma_{R}^{\left(1\right)}}{NV}}$	$B^{\left(\mathrm{SR}\right)}=1$	Non-Res: $∼10^{-9}-10^{-8}$ and Res: $∼10^{-6}-10^{-5}$
1PA	$\alpha^{\left(1\mathrm{PA}\right)}=i\frac{\sigma_{a}^{\left(1\right)}}{k_{j}}$	$B^{\left(1\right)}=1$	1-mM chromophore: VIS/UV: $∼5\times (10^{-5}-10^{-3})$, mid-IR: $∼5\times (10^{-7}-10^{-5})$, and near-IR: $∼5\times 10^{-10}$
SHG	$\alpha^{\left(\mathrm{SHG}\right)}=\beta$	$B^{\left(\mathrm{SHG}\right)}=\sqrt{\frac{2g^{\left(2\right)}}{\varepsilon_{0}c}\frac{p_{i}}{A_{b}}}$	Collagen (tissue): $7.4\times 10^{-5}\;{\mathrm{mW}}^{-1/2}/\mu m$, $6.7\times 10^{-4}$
THG	$\alpha^{\left(\mathrm{THG}\right)}=\gamma$	$B^{\left(\mathrm{THG}\right)}=\frac{2}{\varepsilon_{0}c}\sqrt{\frac{g^{\left(3\right)}}{n_{b}^{3}}}\frac{p_{i}}{A_{b}}$	Triglycerides (cellular): $1.6\times 10^{-5}\;\mathrm{mW}/\mu m^{2}$, $1.3\times 10^{-3}$
CARS/CSRS	$\alpha^{\left(\mathrm{CARS}/\mathrm{CSRS}\right)}=\gamma_{r}$	$B^{\left(\mathrm{CARS}/\mathrm{CSRS}\right)}=\frac{2}{\varepsilon_{0}c}\sqrt{\frac{g^{\left(3\right)}}{n_{b}^{3}}}\frac{\sqrt{p_{p}p_{S}}}{A_{b}}$	1-mM acetonitrile: $-i5.7\times 10^{-7}\mathrm{mW}/\mu m^{2}$, $-i4.6\times 10^{-5}$
SRL/SRG	$\alpha^{\left(\mathrm{SRL}\right)}=\gamma_{r}$, $\alpha^{\left(\mathrm{SRG}\right)}={\gamma_{r}}^{*}$	$B^{\left(\mathrm{SRL}/\mathrm{SRG}\right)}=\frac{2}{\varepsilon_{0}c}\sqrt{\frac{g^{\left(3\right)}}{n_{b}^{3}}}\frac{p_{S/p}}{A_{b}}$	1-mM acetonitrile: $-i5.7\times 10^{-7}\;\mathrm{mW}/\mu m^{2}$, $-i4.6\times 10^{-5}$
ISRS	α(ISRS)=Im{γr}	$B^{\left(\mathrm{ISRS}\right)}=\frac{2}{\varepsilon_{0}c}\sqrt{\frac{g^{\left(3\right)}}{n_{b}^{3}}}\frac{\Gamma_{v}}{\nu_{r}}\frac{p_{\mathrm{pu}}}{A_{b}}$	1-mM acetonitrile: $-i2.1\times 10^{-8}\;\mathrm{mW}/\mu m^{2}$, $-i1.7\times 10^{-6}$
TA/SE	$\alpha^{\left(\mathrm{TA}/\mathrm{SE}\right)}=\Delta \alpha$	$B^{\left(\mathrm{TA}/\mathrm{SE}\right)}=\frac{\eta_{\mathrm{pu}}}{1+\eta_{\mathrm{pu}}+\eta_{\mathrm{se}}}$	1-mM eGFP: TA: $(4.4+i3.0)\times 10^{-4}$ and SE: $-(6.5+i3.0)\times 10^{-4}$
PT	$\alpha^{\left(\mathrm{PT}\right)}=\alpha^{\left(1\mathrm{PA}\right)}$	$B^{\left(\mathrm{PT}\right)}=\frac{2}{3}e^{-2}\frac{A_{a}}{A_{b}}\frac{p_{n}}{\lambda \kappa }{\left(\frac{\partial \varepsilon }{\partial T}\right)}_{T_{0}}$	1-mM eGFP: water: $3.2\times 10^{-3}$ and glycerol: $2.2\times 10^{-2}$

The term $\alpha^{\left(j\right)}$ is the single-molecule effective polarizability defined assuming a coherent interaction, which makes the spontaneous Raman concentration dependent, which is just an artifact of the fact that coherence is implied in the definition. Calculations for the effective susceptibility are taken for a concentration at 1 mM. In the calculations, we consider a beam with a center wavelength of λ=510 nm that is focused with a NA = 0.95 objective lens. This beam has is a pulse train with a duration of $\tau_{p}=50\;\mathrm{fs}$ and a repetition rate of $\nu_{r}=62\;\mathrm{MHz}$, and an average power of 10 mW. Abbreviations for these modalities are defined as follows: 1PA, one-photon absorption; SHG, second-harmonic generation; THG, third-harmonic generation; SR, spontaneous Raman; CARS, coherent anti-Stokes Raman scattering; CSRS, coherent Stokes Raman scattering; SRL, stimulated Raman loss; SRG, stimulated Raman gain; ISRS, impulsive stimulated Raman scattering; TA, transient absorption; SE, stimulated emission; PT, photothermal. Typical numbers are calculated based on a pulsed laser with a ∼20  ps duration for harmonic generations and ISRS and for a ∼5  ps duration for other types of pump–probe Raman methods as well as TA/SE and PT (acts as a CW source).

We may separate the label-free optical interactions into two broad categories: coherent and incoherent. In the incoherent case, each molecule contributes to the change in the detected light power independently of the other molecules within the interaction volume. The total signal is proportional to the concentration in the incoherent case. In the coherent case, each molecule is driven in phase within the interaction volume, and thus, the total susceptibility perturbation is proportional to the molecular concentration, which we specify in terms of the number density, N. Within the volume, there are M=NV total molecules that contribute to the label-free signal generation.

A key aspect of the incoherent case is that the phase of scattering or emission from each molecule fluctuates randomly on a time scale that is rapid compared with the detector integration time. This is the case for autofluorescence and spontaneous Raman scattering. In spontaneous Raman scattering, each molecule scatters light inelastically to new optical frequencies through the modulation of the molecular polarizability due to the thermal excitation of molecular vibrational modes. The phase of the vibrational oscillations, including the phase of the scattered light, is a random variation that changes from molecule to molecule. Within V, each molecule will scatter a power of $p_{R}^{\left(1\right)}=\sigma_{R}^{\left(1\right)}I_{0}$. The origin of Raman scattering is a change in the polarizability, $\delta \alpha^{\left(1\right)}=\alpha^{\prime }Q_{v}$, of the molecule with a displacement of the vibrational coordinate, $Q_{v}$, as weak excitation of a vibrational mode is modeled as a harmonic with an amplitude of $Q_{v0}=\sqrt{\hbar /2\Omega_{v}}$ for vibrational frequency $\Omega_{v}$ driven by thermal excitation. The strength of the polarizability modulation is ${\overset{˜}{\alpha }}^{\prime }$. The Raman scattering cross-section of a single molecule is given as $\sigma_{R}^{\left(1\right)}=(k_{1}^{4}/6\pi )|\delta \alpha^{\left(1\right)}{|}^{2}$, where $\varepsilon_{s}$ is the scattered light wave number. This classically derived model must be slightly modified to account for mode occupancy in a quantum scattering picture, and this modification explains the discrepancy between the amplitude of Stokes and anti-Stokes spontaneous Raman scattering.

Because spontaneous Raman scattering is incoherent, the total power scattered is simply $p_{R}=Mp_{R}^{\left(1\right)}$. The effective Raman scattering cross-section for the volume is $\sigma_{R}^{V}=M\sigma_{R}^{\left(1\right)}$. In the case of Raman scattering without resonant enhancement, the effective Raman susceptibility perturbation is purely real, $\delta \varepsilon^{\left(\mathrm{SR}\right)}=k_{j}^{-2}\sqrt{6\pi M\sigma_{R}^{\left(1\right)}}/V=\delta \varepsilon_{\mathrm{eff}}^{\left(\mathrm{SR}\right)}$. Raman scattering interactions are weak, which is reflected in low Raman scattering cross-sections ranging from $\sigma_{R}^{\left(1\right)}∼10^{-31}$ to $10^{-29}\;{\mathrm{cm}}^{2}$.^68^ Tuning the Raman laser near an electronic absorption resonance can increase the cross-sections to $\sigma_{R}^{\left(1\right)}∼10^{-25}$ to $10^{-21}\;{\mathrm{cm}}^{2}$. Thus, although Raman vibrational spectra are extremely valuable, detection at low species concentrations is exceedingly difficult, and the stimulated Raman and field enhancement techniques have been used to help alleviate this difficulty.

Another class of inelastic scattering processes is those of coherent nonlinear scattering in which light at a fundamental frequency $\omega_{1}=2\pi c/\lambda_{1}$ is incident on a molecule. If the amplitude of the incident field is sufficiently large, the induced dipole moment no longer exhibits a linearly proportional response to the applied electric field. This dipole moment is usually expanded as a Taylor series of the form^69^ $\mu =\tilde{\alpha }E+\tilde{\beta }E^{2}+\tilde{\gamma }E^{3}+\ldots$. The quantities $\beta =\tilde{\beta }/\varepsilon_{0}$ and $\gamma =\tilde{\gamma }/\varepsilon_{0}$ are called the first and second hyperpolarizabilities, respectively. Here, we are assuming that the polarizability and hyperpolarizabilities are isotropic, so the complications of tensor algebra need not be invoked. We use a compact notation by introducing $\alpha^{\left(j\right)}$ as a generalized hyperpolarizability. Thus, we may write the induced nonlinear dipole as $\mu =\varepsilon_{0}\sum_{j}\alpha^{\left(j\right)}E^{j}$. The second-order term with j=2 can represent SHG, where $\alpha^{\left(\mathrm{SHG}\right)}=\beta$ is the hyperpolarizability. In the case of j=3, $\alpha^{\left(\mathrm{THG}\right)}=\gamma$ is the second hyperpolarizability, which includes the case of THG and self-phase modulation driven by the electronic contribution, $\gamma_{e}$, and includes stimulated Raman scattering that arises from the use of the vibrationally resonant component, $\alpha^{\left(3\right)}=\gamma_{v}$.

These hyperpolarizabilities produce a nonlinear source term, Q(j)=kj2α(j)Eijϵ^. Here, the wavenumber at the scattered frequency is $k_{j}=\omega_{j}/c=j\omega_{1}$, and the harmonic frequency is $\omega_{j}=j\omega_{1}$. For a set of molecules in the volume, V, at a number density N, the coherent scattering is described by a nonlinear polarization density, $P^{\left(\mathrm{NL}\right)}=\varepsilon_{0}D^{\left(j\right)}\chi^{\left(j\right)}E_{i}^{j}$. The factor $D^{\left(j\right)}$ is a degeneracy parameter with a value determined by the nonlinear interaction. In the volume of our sub-wavelength sphere with a number density N of molecules, the generalized hyperpolarizabilities for SHG and THG are $\alpha^{\left(2\right)}\to \beta =\chi^{\left(2\right)}/2N$ and $\alpha^{\left(3\right)}\to \gamma =\chi^{\left(3\right)}/4N$, respectively. The nonlinear scattering cross-section, $\sigma_{s}^{\left(j\right)}=(2^{j-1}k_{j}^{4}/6\pi n_{b}^{j}(\varepsilon_{0}c{)}^{j-1})|NV\alpha^{\left(j\right)}{|}^{2}$, is defined through the instantaneous scattered power. As nonlinear optical interaction strengths are weak, pulsed lasers are used to ensure a large enough peak field strength to produce sufficient rates of nonlinear scattering.

The total time-averaged power scattered by a nonlinear dipole source with frequency $\omega_{j}$, whether from a single molecule or a distribution inside of a sub-wavelength sphere, is $p_{j}=\sigma_{\mathrm{eff}}^{\left(j\right)}I_{a}$. We defined an effective linear cross-section for the nonlinear scattering process as $\sigma_{\mathrm{eff}}^{\left(j\right)}=\sigma_{s}^{\left(j\right)}g^{\left(j\right)}I_{a}^{j-1}$. Notice that this effective cross-section depends nonlinearly on the average intensity of the incident fundamental beam, $I_{a}$, and on the zero-lag j’th-order intensity correlation function, $g^{\left(j\right)}$. This correlation function, defined as $g^{\left(j\right)}=\langle I^{j}(t)\rangle /{\left\langle I(t)\right\rangle }^{j}$, depends on the duty cycle, $\nu_{r}\tau_{p}$, of the fundamental excitation beam laser source. Although the exact value of $g^{\left(j\right)}$ depends on the pulse shape, the value is bounded by $g^{\left(j\right)}\leq (\nu_{r}\tau_{p}{)}^{-(j-1)}$, where the upper bound is met with a square pulse. The effective cross-section defines an effective linear susceptibility perturbation through the relationship $\delta \varepsilon_{\mathrm{eff}}=\sqrt{6\pi \sigma_{\mathrm{eff}}^{\left(j\right)}}/k_{j}^{2}V$. With this, we define the effective linear susceptibility perturbation for coherent harmonic scattering as $\delta \varepsilon_{\mathrm{eff}}=N\alpha^{\left(j\right)}B^{\left(j\right)}$. The factor $B^{\left(j\right)}=\sqrt{(2/\varepsilon_{0}c{)}^{\left(j-1\right)}g^{\left(j\right)}I_{a}^{\left(j-1\right)}}$ accounts for averaging the nonlinear scattered power over the pulse train. The effective cross-section and susceptibility can be directly used in the signal model given in Eq. (5). This equation also includes the term $\ell^{\left(j\right)}=h^{\left(j\right)}/\sqrt{g^{\left(j\right)}}$. The term $h^{\left(j\right)}=I_{a}^{-(j+1)/2}\langle I(t{)}^{(j+1)/2}\rangle$ arises as an interference factor from signal averaging over the pulse train. This term is also bounded as $h^{\left(j\right)}\leq \sqrt{g^{\left(j\right)}}$, where the bounds are again saturated by a square pulse. In the case of a square pulse, $\ell^{\left(j\right)}=1$.

Single molecules interacting can absorb light through linear or nonlinear absorption processes, and optical absorption can occur for electronic and vibrational energy level transitions. Exactly on resonance, the polarizability for a molecule becomes purely imaginary, i.e., $\alpha^{\left(1\right)}\to i\alpha_{i}^{\left(1\right)}$. The superscript indicates that we are dealing with the polarizability of a single molecule. This polarizability produces both absorption and scattering, with cross-sections for extinction, σe(1)=k0Im{α(1)}; scattering, $\sigma_{s}^{\left(1\right)}=(k_{0}^{4}/6\pi )|\alpha^{\left(1\right)}{|}^{2}$; and absorption, $\sigma_{a}^{\left(1\right)}=\sigma_{e}^{\left(1\right)}-\sigma_{s}^{\left(1\right)}$. As, generally, $\sigma_{a}^{\left(1\right)}\ll \lambda^{2}$, the absorption cross-section on resonance, when $\alpha^{\left(1\right)}$ is purely imaginary, the absorption cross-section is well approximated by $\sigma_{a}^{\left(1\right)}\approx k_{0}\alpha_{i}^{\left(1\right)}$. The perturbation to the linear susceptibility from a molecule number density of N is then $\delta \varepsilon_{\mathrm{eff}}^{\left(\mathrm{abs}\right)}=iN\alpha_{i}^{\left(1\right)}\approx iN\sigma_{a}^{\left(1\right)}/k_{0}$. The absorption cross-sections vary over a wide range, with a maximum value on the order of $\sigma_{a}∼\lambda^{2}/2$, which corresponds to $\delta \varepsilon_{\mathrm{abs}}=iN\alpha_{i}^{\left(1\right)}\approx iN\lambda^{3}/4\pi$. Chromophores have absorption cross-sections ranging from^68^ $\sigma_{a}^{\left(1\right)}∼10^{-17}$ to $10^{-15}\;{\mathrm{cm}}^{2}$ for visible and ultraviolet absorption. These numbers drop several orders of magnitude for mid-infrared vibrational spectra that exhibit cross-sections in the range of $\sigma_{a}^{\left(1\right)}∼10^{-19}$ to $10^{-17}\;{\mathrm{cm}}^{2}$. Overtone stretches are generally weaker, on the order of range $\sigma_{a}^{\left(1\right)}∼10^{-22}$.^70^^,^^71^

Another common absorption mechanism is multiphoton absorption, in which the promotion of an electron from a ground to an excited state requires the simultaneous arrival of two or more photons with energy below the energy gap. For degenerate two-photon absorption, the interaction of the fields induces an instantaneous perturbation to the effective linear optical susceptibility of^72^ $\delta \varepsilon_{2\mathrm{PA}}=(3/4)\chi^{\left(3\right)}|E_{i}{|}^{2}$. This perturbation is complex-valued, indicating that both the self-phase modulation and the two-photon absorption are driven in this interaction. Moreover, the existing linear susceptibility dominates this interaction, i.e., $\chi^{\left(1\right)}\gg \delta \varepsilon_{2\mathrm{PA}}$, and thus, the change in field strength and the extinguished power are vanishingly small for two-photon absorption. As a result, two-photon and multiphoton absorption in general are typically used with efficient fluorophores, in which emitted fluorescent light is collected as the signal. Thus, we do not discuss the direct detection of molecules through multiphoton absorption in the context of direct detection.

The limitations of spontaneous Raman scattering can be partially mitigated using stimulated Raman methods. These techniques are nonlinear optical methods in which a two-photon resonant excitation is driven at the vibrational frequency, $\Omega_{v}$, in a molecule. The stimulated two-photon process is driven by two incident fields, a pump field, $E_{p}$, at frequency $\omega_{p}$ and a Stokes field, $E_{S}$, at frequency $\omega_{S}<\omega_{p}$. At resonance, the frequency difference is set to $\omega_{p}-\omega_{S}=\Omega_{v}$. There are many subtleties in dealing with the description of stimulated Raman scattering, and we focus on the vibrationally resonant part of the nonlinear optical response arising from $\chi_{\mathrm{VR}}^{\left(3\right)}$. However, the presence of nonlinear phase modulation from the electronic contribution to the nonlinear optical susceptibility presents challenges and opportunities—depending on the experimental arrangement.

The CARS and CSRS nonlinear scattering processes also coherently produce light at a new optical frequency of $\omega_{\mathrm{aS}/\mathrm{cS}}$, where aS and cS denote the anti-Stokes and Stokes frequencies, respectively. We define an effective susceptibility that may be computed in an analogous manner to the case of SHG and THG scattering, resulting in $\delta \varepsilon_{\mathrm{eff}}^{\left(\mathrm{CARS}/\mathrm{CSRS}\right)}=NB^{\left(\mathrm{CARS}/\mathrm{CSRS}\right)}\gamma^{\left(CRS\right)}$. Here, $B^{\left(\mathrm{CARS}/\mathrm{CSRS}\right)}=(2/\varepsilon_{0}c)\sqrt{g^{\left(\mathrm{CARS}/\mathrm{CSRS}\right)}I_{\mathrm{ap}}I_{\mathrm{aS}}}$, which depends on the product of the average power of the pump and Stokes beams. Here, we also assumed that the temporal profile of the pump and Stokes pulses are identical, so $g^{\left(\mathrm{CARS}/\mathrm{CSRS}\right)}=g^{\left(3\right)}$. The same is true for $h^{\left(\mathrm{CARS}/\mathrm{CSRS}\right)}=h^{\left(3\right)}$. The explicit expression for the effective scattering cross-section now reads as $\sigma_{\mathrm{eff}}^{\left(\mathrm{CARS}/\mathrm{CSRS}\right)}=(2k_{\mathrm{aS}/S}^{4}V^{2}/3\pi n_{b}^{3}(\varepsilon_{0}c{)}^{2})|\delta \varepsilon_{\mathrm{eff}}^{\left(\mathrm{CARS}/\mathrm{CSRS}\right)}{|}^{2}$. The wavenumbers are given by $k_{\mathrm{aS}/\mathrm{cS}}=\omega_{\mathrm{aS}/\mathrm{cS}}/c$, and the SRS hyperpolarizability reads as $\gamma^{\left(\mathrm{CRS}\right)}=(6/4N)\chi^{\left(3\right)}(\Omega_{v})$. Because the CARS and CSRS processes are driven by two fields, the expression for the averaged scattered power depends on the process as $p_{\mathrm{aS}/\mathrm{cS}}=\sigma_{\mathrm{eff}}^{\left(\mathrm{CARS}/\mathrm{CSRS}\right)}I_{p/S}$.

A set of label-free interactions falls into the category of pump–probe interactions, which are distinguished by the excitation of a non-equilibrium condition in the system by a first (pump) pulse. The non-equilibrium condition evolves with time and produces a time-varying change in the optical properties of the system that is probed by a second pulse (probe) that arrives at a later time. The excitation by the pump pulse produces a perturbation in the effective linear susceptibility, $\delta \varepsilon_{\mathrm{eff}}(t)$ for t>0, where we denote t=0 as the arrival time of the pump pulse. The time dependence of the susceptibility perturbation produces spectral scattering that slightly modifies the detected signals. We neglect the spectral scattering effects, but a recent review of coherent Raman scattering analyzes this scenario in detail.^42^

The non-equilibrium condition may be established by the rearrangement of the population among electronic, vibrational, or rotational energy levels. Following the perturbation of the system, the kinetics of the relaxation of the excited state dictates perturbations to the optical properties of the system that can be detected by a time-delayed probe pulse. Although these subsequent dynamics can be quite complicated, the effect on the probe pulse can be modeled by a complex-valued $\delta \varepsilon_{\mathrm{eff}}(t)$, and the details of the description depend on the detailed spectroscopy interrogated by the probe pulse, which can be tuned in wavelength to vary the interaction dynamics.

In the case of optical absorption induced by a pump pulse, the pump pulse moves the population density from the ground to an excited electronic state by a change in number density $\delta N=Ne_{m}$. Here, N is the population density of the molecules, and $e_{m}=\eta_{\mathrm{pu}}/(1+\eta_{\mathrm{pu}}+\eta_{\mathrm{se}})$ is the average excitation probability of a molecule subject to the pump field. We included the competing processes of stimulated absorption that is used to excite the molecule, with an excitation parameter $\eta_{\mathrm{pu}}=I_{\mathrm{pu}}/I_{\mathrm{sat}}$. The related stimulated emission parameter is $\eta_{\mathrm{se}}=I_{\mathrm{pr}}/I_{\mathrm{se}}$. The efficiency of excitation depends on the pump intensity relative to the saturation intensity, $I_{\mathrm{sat}}=\hbar \omega_{\mathrm{pu}}/\tau_{e}\sigma_{a}$. Similarly, the efficiency of simulated de-excitation, i.e., emission, depends on the probe intensity relative to the stimulated emission intensity, $I_{\mathrm{se}}=\hbar \omega_{\mathrm{pr}}/\tau_{e}\sigma_{\mathrm{se}}$.

This population transfer admits several optical spectroscopy perturbations for a time-delayed probe pulse. Details of the particular spectroscopic interactions depend on the center wavelength of the probe pulse. Absorption of the probe pulse can be reduced through ground state depletion or increased through excited state absorption, processes referred to as TA. Alternatively, at some probe wavelengths, a population inversion can be established, leading to SE that amplifies the probe pulse.^25^^,^^27^^,^^50^^,^^52^ The change in susceptibility following pump pulse excitation is given by $\delta \varepsilon_{\mathrm{eff}}^{\left(\mathrm{TA}/\mathrm{SA}\right)}=\Delta \alpha^{\left(1\right)}\delta N$, for the change in the single-molecule polarizability between the ground and excited states at the probe wavelength, $\Delta \alpha^{\left(1\right)}$. In general, $\Delta \alpha^{\left(1\right)}=\Delta \alpha_{r}^{\left(1\right)}+i\Delta \alpha_{i}^{\left(1\right)}$ is complex-valued. When dominated by the imaginary component, $\Delta \alpha_{i}^{\left(1\right)}$, this process is called TA for positive values and SE for negative values. When the real component, $\Delta \alpha_{r}^{\left(1\right)}$, dominates, the population change is detected through a phase modulation. In all cases, the susceptibility perturbation drives a change in the scattering from the molecule.^52^ The signal change to the probe pulse causes either gain or loss in the probe field. For the signal model in Eq. (5), TA and SE make use of the scattering cross-sections from the volume given by $\sigma_{\mathrm{eff}}^{\left(\mathrm{TA}/\mathrm{SE}\right)}=(k_{\mathrm{pr}}^{4}V^{2}/6\pi )|\delta \varepsilon_{\mathrm{eff}}^{\left(\mathrm{TA}/\mathrm{SA}\right)}{|}^{2}=\sigma_{\mathrm{eff}}^{\left(\mathrm{TA}/\mathrm{SE}\right)}$. The wavenumber of the probe pulse is $k_{\mathrm{pr}}=\omega_{\mathrm{pr}}/c$, and $B^{\left(\mathrm{TA}/\mathrm{SE}\right)}=1$, $g^{\left(\mathrm{TA}/\mathrm{SE}\right)}=1$, and $h^{\left(\mathrm{TA}/\mathrm{SE}\right)}=1$.

As noted above, the change in population δN is a perturbation away from thermal equilibrium. This change in population density for a probe pulse with a fluence well below saturation can be computed for a square pulse with peak intensity $I_{0}$ and pulse duration $\tau_{p}$, with δN=ρN and parameter $e_{m}=\tau_{p}\lambda I_{0}\sigma_{a}^{\left(1\right)}/hc$, where h is Planck’s constant. In the case of two-photon absorption, $e_{m}=\tau_{p}\lambda I_{0}^{2}\sigma_{2\mathrm{PA}}^{\left(1\right)}/hc$, where $\sigma_{2\mathrm{PA}}^{\left(1\right)}$ is the 2PA cross-section for a single molecule in units of $m^{2}/W$. This excited state excitation will relax back to the ground state to reach thermal equilibrium. Although this energy decay is often described by a single exponential decay with an excited state lifetime, $\tau_{e}$, on the order of a few picoseconds for nonfluorescent chromophores, the decay dynamics vary across molecular systems and can be extremely complicated. In addition, this relaxation will lead to the thermalization of energy deposited in the molecule with the surrounding environment that can also be used for label-free imaging through PT detection, as described below.

Pump–probe interactions are also used for vibrational spectroscopic measurements. Although vibrational effects, and thus vibrational spectroscopy, can be extracted from dynamics on the excited state of molecules, in label-free microscopy, vibrational spectroscopy is usually probed on the ground state through SRS. The processes of SRS are driven by pulses overlapped in time and produce multiple processes that occur at the same time as CARS, CSRS, and SRS scattering. SRS, produces both loss at the pump frequency driven by the intensity of the Stokes field, leading to stimulated Raman loss (SRL), and gain at the Stokes frequency, leading to stimulated Raman gain (SRG).

Both SRL and SRG produce an effective instantaneous linear susceptibility change that may be written as $\delta \epsilon_{\mathrm{eff}}^{\left(\mathrm{SRL}\right)}=NB^{\left(\mathrm{SRL}\right)}\gamma^{\left(\mathrm{CRS}\right)}$ and $\delta \epsilon_{\mathrm{eff}}^{\left(\mathrm{SRG}\right)}=NB^{\left(\mathrm{SRG}\right)}\gamma^{(\mathrm{CRS})*}$. Here, $B^{\left(\mathrm{SRL}/\mathrm{SRG}\right)}=(2/\varepsilon_{0}c)\sqrt{g^{\left(3\right)}}I_{\mathrm{aS}/\mathrm{ap}}$, which depends on the product of the average power of either the pump or Stokes beam. The average SRG and SRL power scattered is $p_{\mathrm{SRG}/\mathrm{SRL}}=\sigma_{\mathrm{eff}}^{\left(\mathrm{SRL}/\mathrm{SRG}\right)}I_{p/S}$. Because the vibrationally resonant contribution to the third-order susceptibility is purely imaginary at peak excitation, $\chi^{\left(3\right)}∼i(N\varepsilon_{0}/12m\Omega_{v}\Gamma_{v}){\left(\alpha^{1}\right)}^{2}∼i3\times 10^{-20}\;m^{2}/V^{2}$, $\delta \epsilon_{\mathrm{eff}}^{\left(\mathrm{SRL}/\mathrm{SRG}\right)}$ is purely imaginary and thus behaves analogously to TA for SRL and SE for SRG.^41^ Here, m is the reduced mass of the vibrational mode, and $\Gamma_{v}$ is the line width of the vibrational mode resonance. SRL and SRG modify the pump and the Stokes beams through absorption and scattering, where $\alpha^{\left(\mathrm{SRL}\right)}=\gamma^{\left(\mathrm{SRS}\right)}$ and $\alpha^{\left(\mathrm{SRG}\right)}=\gamma^{(\mathrm{SRS})*}$. Finally, we note that $B^{\left(\mathrm{SRL}/\mathrm{SRG}\right)}=(2/\varepsilon_{0}c)\sqrt{g^{\left(3\right)}}I_{\mathrm{aS}/\mathrm{ap}}$.

SRS is usually implemented with laser pulses longer than the decay time of the excited vibrational coherence. When a pulse duration is shortened so that the $\tau_{p}\Omega_{v}\ll 1$, the vibrations are driven impulsively by drawing pump and Stokes frequencies from within the bandwidth of a single pump pulse. This limit is referred to as ISRS.^42^ A vibrational coherence is prepared in the molecule following interaction with a short pump pulse. This vibrational coherence in the impulsive excitation limit produces an oscillating polarizability that leads to an optical susceptibility perturbation of δεeff(ISRS)=NB(ISRS) Im{γ(r)}. In the impulsive case, we have $B^{\left(\mathrm{ISRS}\right)}=(3/\varepsilon_{0}c)(\Gamma_{v}/\nu_{r})I_{a,\mathrm{pu}}$, where $I_{a,\mathrm{pu}}=p_{\mathrm{pu}}/A_{\mathrm{pu}}$ denotes the average intensity of the pump pulse and $\Gamma_{v}$ is the decay rate of the vibrational coherence. As with TA/SE, a time-delayed probe pulse interacts with the susceptibility perturbation to produce linear scattering from a spherical particle with polarizability $\alpha^{\left(\mathrm{ISRS}\right)}=V\delta \varepsilon^{\left(\mathrm{ISRS}\right)}$ and with the usual scattering cross-section, so we use $p_{\mathrm{pr}}$ for Eq. (5).

The final pump–probe interaction that we consider is the PT effect in which a local change in temperature leads to a change in the local optical susceptibility, $\delta \varepsilon_{\mathrm{eff}}^{\left(\mathrm{PT}\right)}=\Delta T(\partial \varepsilon /\partial T)$, that modifies linear scattering for a probe pulse identical to the cases of any optical excitation process. The induced perturbation depends on thermal transport because the susceptibility change depends on the change in temperature, ΔT, that is driven by heating from energy deposited into the system. The heating and thermal distribution are dictated by the heat capacity and thermal transport properties of the medium, respectively. As we are considering a system in which the target of unlabeled molecules is confined in the volume of a sphere with radius a≪λ, we model the optical response with point heating. On timescales shorter than the thermal transport time, we estimate an upper bound on the local temperature rise from the energy deposited per excitation $E_{\mathrm{ex}}=\hbar \Omega_{\mathrm{ex}}$, where $\Omega_{\mathrm{ex}}$ is the energy gap between ground and excited states. These states can be electronic^73^^–^^76^ or vibrational.^57^^,^^58^ The total change in energy for $M_{\mathrm{ex}}$ states is given as $\Delta Q=E_{\mathrm{ex}}M_{\mathrm{ex}}\eta_{\mathrm{nr}}$. The temperature rise from the heating by the thermal relaxation to the surroundings of the energy deposited in the excitations within a volume, V, is $\Delta T=\Delta Q/C_{v}V$, where $C_{v}$ is the heat capacity per unit volume of the solvent surrounding the absorber. Nonradiative relaxation of this energy leads to local heating, producing the change in optical susceptibility that is exploited by PT detection. For the case of linear optical absorption, on average, the efficiency of nonradiative relaxation $\eta_{\mathrm{nr}}=k_{\mathrm{nr}}/(k_{\mathrm{nr}}+k_{r})=1-\Phi_{f}$, which is the complement to the quantum fluorescent yield of a molecule, determines the fraction of the energy absorbed by the molecule that contributes to heating. Thus, non-fluorescing chromophores are the best candidates for PT detection. The nonradiative and radiative relaxation rates are $k_{\mathrm{nr}}$ and $k_{r}$, respectively. The excited state lifetime of a molecule, given by $\tau_{e}=(k_{\mathrm{nr}}+k_{r}{)}^{-1}$ and which is on the order of several picoseconds for non-fluorescing molecules or several nanoseconds for fluorescent molecules, sets the times scales for the population kinetics following excitation.

The thermal timescales in a biological imaging scenario are dominated by conductive heat transport. Conductive thermal transport is modeled by the diffusion equation, which gives a diffusion radius $L_{\mathrm{th}}=\sqrt{4Dt}$ in an infinite thermal medium, where t is the time after the point heating has occurred. The thermal transport of the heat away from the absorbers depends on the thermal conductivity, $\kappa =DC_{v}$, and the diffusion coefficient, D. As we are considering the detection of sub-wavelength particles, we can establish a thermal time scale for diffusion over a wavelength, set by $t_{\mathrm{th}}=\lambda^{2}/4D$. Using a typical value for the diffusion coefficient, $D∼10^{-6}\;m^{2}/s$, and $\lambda =10^{-6}\;m$, we obtain $t_{\mathrm{th}}=25\;\mu s$. This timescale is much larger than the pulse spacing in a typical mode-locked oscillator, $t_{\mathrm{th}}\gg \nu_{r}^{-1}$, so we may treat the heating and detection with the average beam powers. To eliminate the effects of stray background absorption and scattering, the heating beam is modulated with frequency $\nu_{\mathrm{mod}}$ and the thermal transport length associated with this modulation frequency gives a radius of $r_{\mathrm{th}}=\sqrt{D/\pi \nu_{\mathrm{mod}}}$, which we consider for defining an effective volume of the heated region that induces scattering on the probe pulse.

On a time interval Δt shorter than $t_{\mathrm{th}}$, where $\Delta t\ll t_{\mathrm{th}}$, there is little time for heat to diffuse as $r_{\mathrm{th}}$ will be much less than λ. However, given such short times, we can estimate the heating that is produced by $M_{p}=\Delta t\nu_{r}$ pulses in the time interval for a pulsed source with a repetition frequency of $\nu_{r}$. The heating per pulse, ΔQ, will accumulate to a total temperature rise of $\Delta T=\Delta QM_{p}/C_{v}V$. For optical absorption well below saturation, the mean number of molecules excited by a square heating pulse of length $\tau_{h}$ and peak intensity $I_{0h}$ is given by $M_{\mathrm{ex}}=\tau_{h}\sigma_{a}^{\left(1\right)}I_{0h}NV/E_{\mathrm{ex}}$. Putting this together, we obtain a susceptibility perturbation of $\delta \varepsilon_{\mathrm{eff}}^{\mathrm{PT}}=N\delta \varepsilon_{i}^{\left(1\right)}B_{\mathrm{PT}}$. Here, the imaginary component of the single-molecule susceptibility is $\delta \varepsilon_{i}^{\left(1\right)}=\sigma_{a}^{\left(1\right)}k_{0}^{-1}$, and the photothermal factor is $B_{\mathrm{PT}}=(2/3)e^{-2}(A_{a}/A_{h})(\Delta t_{h}p_{h}/\lambda \kappa )(\partial \varepsilon /\partial T{)}_{T_{0}}$. The heating depends on the ratio of the sphere cross section area, $A_{a}=\pi a^{2}$, to the heating beam cross-section area, $A_{h}$, and the ratio of the average power of the heating beam, $p_{h}$, to the product λ κ. Heating is converted into a change in optical susceptibility through $(\partial \varepsilon /\partial T{)}_{T_{0}}$ at an equilibrium temperature $T_{0}$. The thermal properties of the solvent that scale the PT susceptibility perturbation are $\kappa^{-1}(\partial \varepsilon /\partial T{)}_{T_{0}}$. Using the numbers from the literature,^77^ we find that this figure of merit is ∼6.6× larger for glycerol than water, which is why PT imaging experiments use glycerol as a solvent when possible.^59^

## 4 Single-Pixel Detection of Different Label-Free Imaging Methods

It is evident upon inspection of Eq. (5) that many experimental modalities are admitted by this expression. Having established the general model for the estimation of either a real- or imaginary-valued optical susceptibility perturbation, we now study the relative performance of methods and comment on the detection sensitivity of various optical methods. We begin with the case of direct signal detection without the aid of interferometric enhancement that is enabled by mixing with a coherent reference field. With this baseline established, we examine the benefit of reducing noise power via the elimination of the incident field in detection, alternatively known as dark-field imaging.

Although experimentally challenging due to the potential weakness of the signal, dark-field imaging oftentimes utilizes a detection direction different from the incident. The normalized Fisher information, for both cases of purely real- and imaginary-valued susceptibilities, is expressed as

$${\overset{˜}{J}}_{\mathrm{df}}=4\Gamma =\eta_{c}\frac{k_{0}^{4}V^{2}}{3\pi A_{b}}.$$ (10)

It is independent of the object susceptibility and increases as the object volume increases or as the incident beam area decreases. The key factor in dark-field detection is the collection efficiency. A small collection efficiency will produce a weak photon flux, and if this flux drops below the rate of dark current detection or background flux incident on the detector, long integration times are required. Similarly, when we consider the interaction volume, V, a small volume also makes dark-field detection weak.

Bright-field imaging, by contrast, is a more commonly employed technique in optical experiments. However, the presence of the incident field, whether or not it interferes with the object field, leads to an elevation in the noise level. Increased noise decreases the Fisher information, and this is more evident in the real-valued case thanks to the absence of interference between the incident and scattered fields. The corresponding normalized Fisher information expression is given by

$${\overset{˜}{J}}_{\mathrm{bf}}\approx \{\begin{array}{ll} 4\Gamma^{2}(\delta \varepsilon_{\mathrm{eff}}^{\left(\mathrm{re}\right)}{)}^{2} & \text{real-valued }\delta \epsilon_{\mathrm{eff}}\\ H^{2} & \text{imaginary-valued }\delta \epsilon_{\mathrm{eff}} \end{array}.$$ (11)

We observe a drastic difference in behavior between the cases in which the susceptibility takes on a real or imaginary value. In the bright-field case, the background light passes the sample and hits the detector, along with the scattered field. When the susceptibility is real-valued, there is no energy exchange with the molecules to be detected; rather, light is scattered from the scatterer and is shifted in phase by π/2. Because of this phase shift, the scattered field does not interfere with the (much stronger) unscattered field. As a result, the detected signal is extremely weak compared with the noise, which is dominated by the unscattered beam. By contrast, if the susceptibility is imaginary-valued, there will be a change in the field strength of the light passing through the object that is small, but this altered field will still interfere with the large unscattered field, which amplifies the signal and provides more information relative to noise. Clearly, the scattering phase shift makes the detection of real-valued susceptibilities much more difficult than the imaginary case. This situation sharply contrasts with the scenario for dark-field detection in which either susceptibility perturbation value leads to the same normalized Fisher information.

In the case of interference with a dark-field signal, we obtain the anticipated result in the limit of a large reference beam for which the normalized fisher information reaches the case of bright-field where interference between the scattered and incident light can occur:

$${\overset{˜}{J}}_{\mathrm{df},\mathrm{int}}\approx \{\begin{array}{ll} H^{2}\;\sin^{2}\;\phi & \text{real-valued }\delta \epsilon_{\mathrm{eff}}\\ H^{2}\;\cos^{2}\;\phi & \text{imaginary-valued }\delta \epsilon_{\mathrm{eff}} \end{array}.$$ (12)

This result relies on relative coherence between the scattered and reference fields. With that coherence comes an additional degree of freedom in the experiment, the relative phase ϕ. When ϕ is suitably chosen, we reach the limiting case of bright-field detection, regardless of whether the susceptibility is real or imaginary.

Finally, we have the general scenario for the Fisher information. It is helpful to consider two situations: (a) the limit of a very strong reference field and (b) when we reach an interferometric null, i.e., with a, R=1 and the correctly chosen phase, ϕ=π. In the case of a strong reference field, the exact same result as for the dark-field case given in Eq. (12) is obtained. In the interferometric null case, we zero out the incident field with the reference field at the detector, eliminating the noise contribution from the background light as well. Under this condition, the Fisher information is identical to the dark-field condition, 4Γ. Another common scenario is to set the reference to a quadrature phase, where ϕ=±π/2, in which case, the normalized Fisher information is half the value for bright-field $H^{2}/2$.

The question now arises as to how to arrange an experimental configuration to optimize the detection sensitivity of a target molecule and thus to reach the smallest value of $\delta \epsilon_{\mathrm{eff}}$ that is contained within a volume V. This formulation was chosen because optical label-free detection at single-molecule sensitivity is usually infeasible, so we must consider an interaction region that contains some concentration of the target. The preceding analysis provides us with two distinct limiting cases that bound the available per-photon normalized Fisher information: the dark- and bright-field cases; the ratio of these is given by

$$\rho_{N}=\frac{{\overset{˜}{J}}_{\mathrm{df}}}{{\overset{˜}{J}}_{\mathrm{bf}}}=\frac{4\Gamma }{H^{2}}=\frac{\eta_{c}k_{0}^{2}A_{b}}{3\pi (\ell f_{\mathrm{NA}}{)}^{2}}.$$ (13)

It is interesting to note that the relative signal size does not depend on the sample volume. For a simple estimate of this ratio, we may set the beam area to $A_{b}=\lambda^{2}$, and noting that ℓ and $f_{\mathrm{NA}}$ are on the order of unity, we reach the approximation $\rho_{N}\approx (4\pi /3)\eta_{c}∼4\eta_{c}$. When $\rho_{N}>1$, dark-field detection contains a higher normalized Fisher information than the bright-field case, which could occur with sufficiently large collection efficiency. Using the full expression for $A_{b}$, $\eta_{c}$, and $f_{\mathrm{NA}}$, we find that, above an NA of 0.62, the collection efficiency is high enough that the normalized Fisher information is larger than the bright-field case. It should be noted that this does not imply a larger dark-field signal but rather that there is substantially less noise from the target object in the case of dark-field detection.

**Fig. 2:**
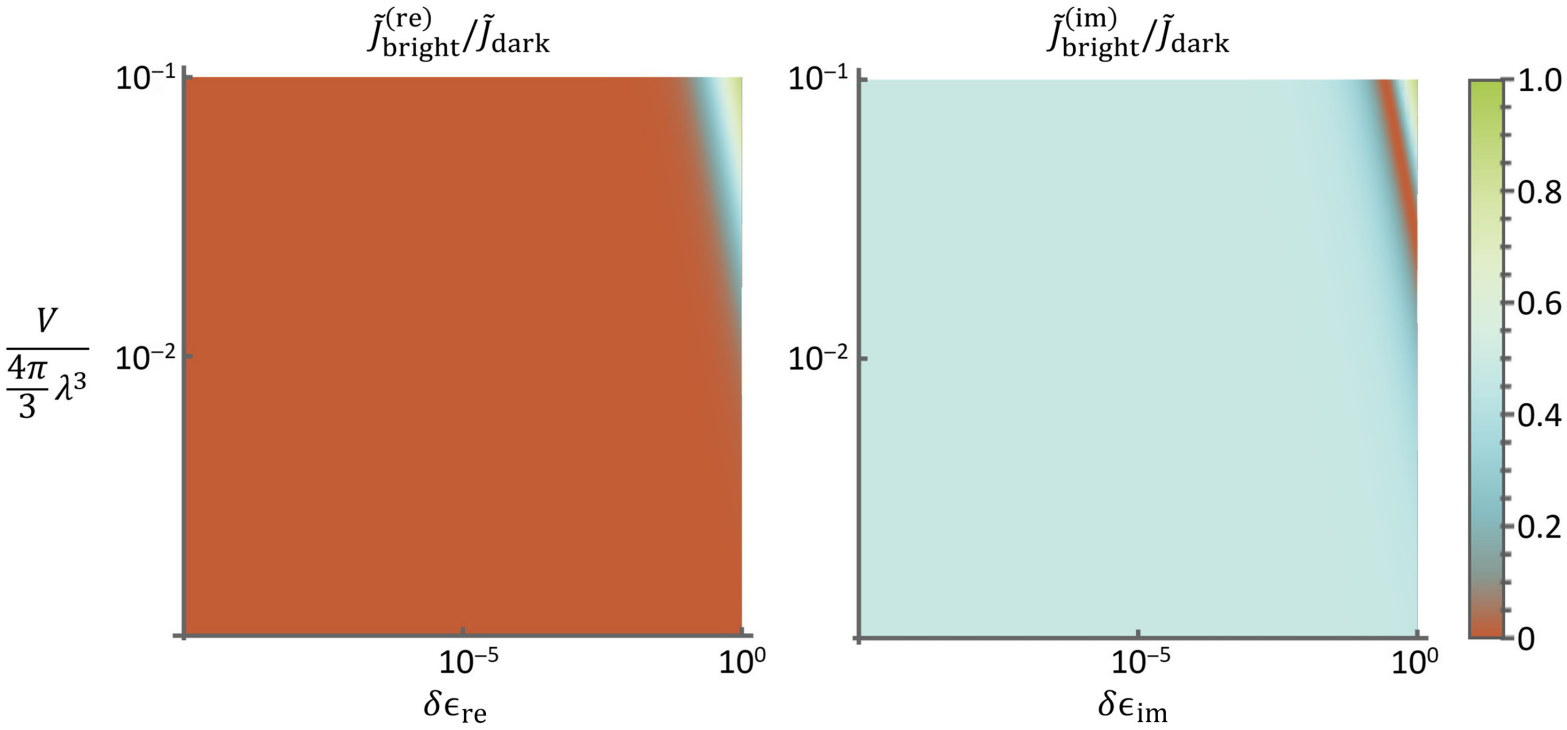
Ratio between the normalized Fisher information in bright-field imaging, ${\tilde{J}}_{\mathrm{bf}}$, and in dark-field imaging or imaginary-valued ($\delta \varepsilon_{\mathrm{im}}$) case, ${\tilde{J}}_{\mathrm{df}}$, is plotted as a function of the object susceptibility, for either a real-valued ($\delta \varepsilon_{\mathrm{re}}$) case and volume, $V=4\pi a^{3}/3$, with a sample spherical volume with radius a, for the case in which no reference field is present; thus, no interference is contained in the recorded photon counts. Recall that this is the Fisher information normalized to total detected photon counts and thus is focused on information obtained in the detection strategy. Here, the NA of the objective is large enough that dark-field detection leads to a larger normalized Fisher information. Due to the lack of inference in the case in which the susceptibility is purely real, the Fisher information remains extremely weak, except for very strong scattering when the volume and susceptibility perturbation become large. By contrast, the case of a purely imaginary susceptibility provides a moderate normalized Fisher information relative to the dark-field case for a wide range of scenarios.

The broader behavior of the normalized Fisher information can be studied by plotting the ratio of the full dark-field and bright-field expressions. The case with no reference beam, and thus no interference, is shown in Fig. 2. With an increase in the strength of the object signal—whether due to a higher object susceptibility and/or a larger volume—the normalized Fisher information approaches that in dark-field imaging, as illustrated in Fig. 2. For values of optical susceptibility and volume typical of experimental systems, the normalized Fisher information in bright-field imaging never exceeds approximately ≪1% of that in dark-field imaging for purely real susceptibility perturbation and ∼50% for purely imaginary susceptibility perturbation.

**Fig. 3:**
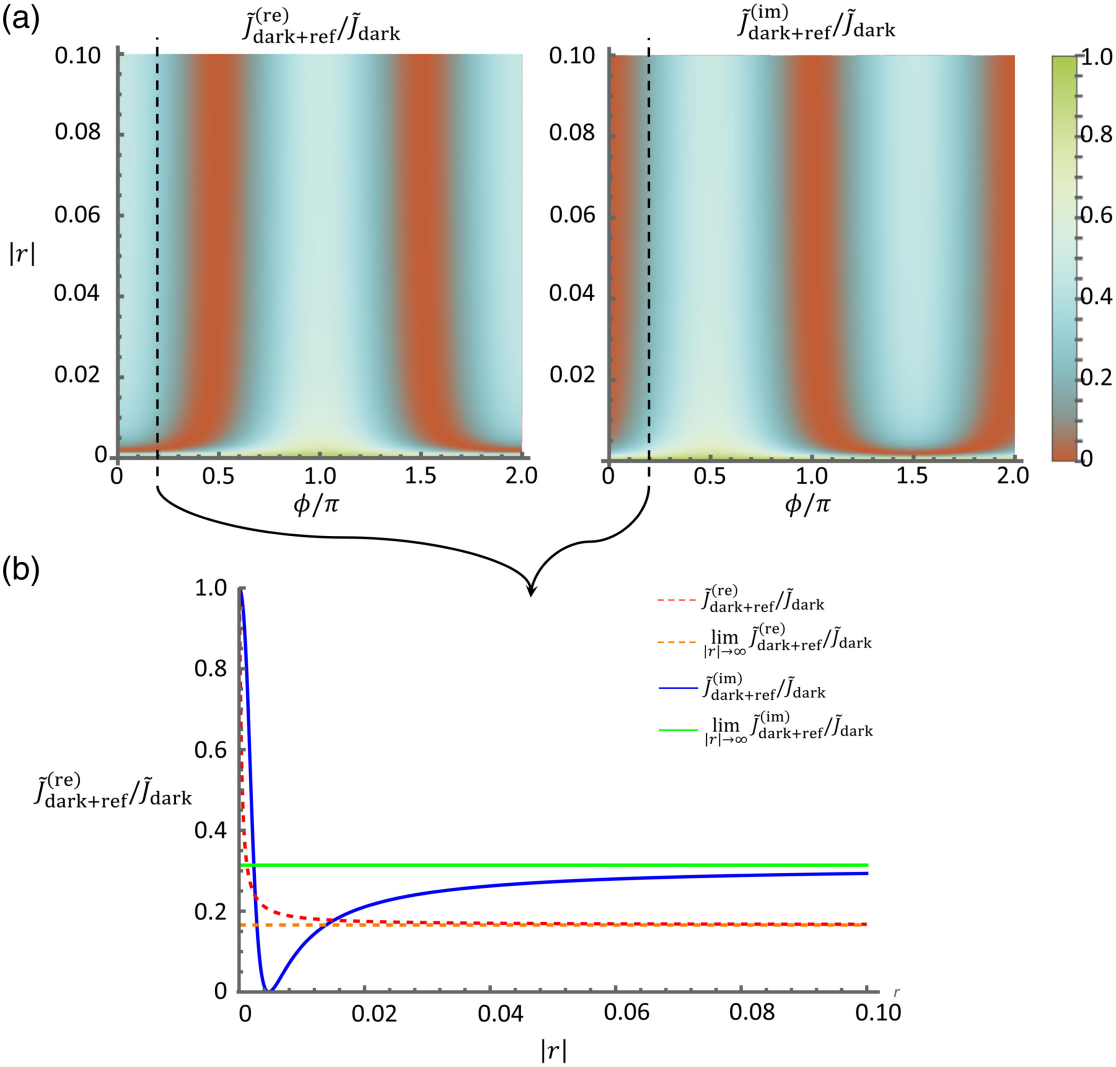
Ratio between the normalized Fisher information in dark-field imaging with reference, ${\tilde{J}}_{\mathrm{df},\mathrm{int}}$, and dark-field imaging, ${\tilde{J}}_{\mathrm{df}}$. (a) Plotted as a function of the relative amplitude, R, and phase, ϕ, of the reference field. (b) Plot this ratio for a fixed phase value as the function of the amplitude of the reference to demonstrate its asymptotic behavior compared with the corresponding approximated limit expression.

**Fig. 4:**
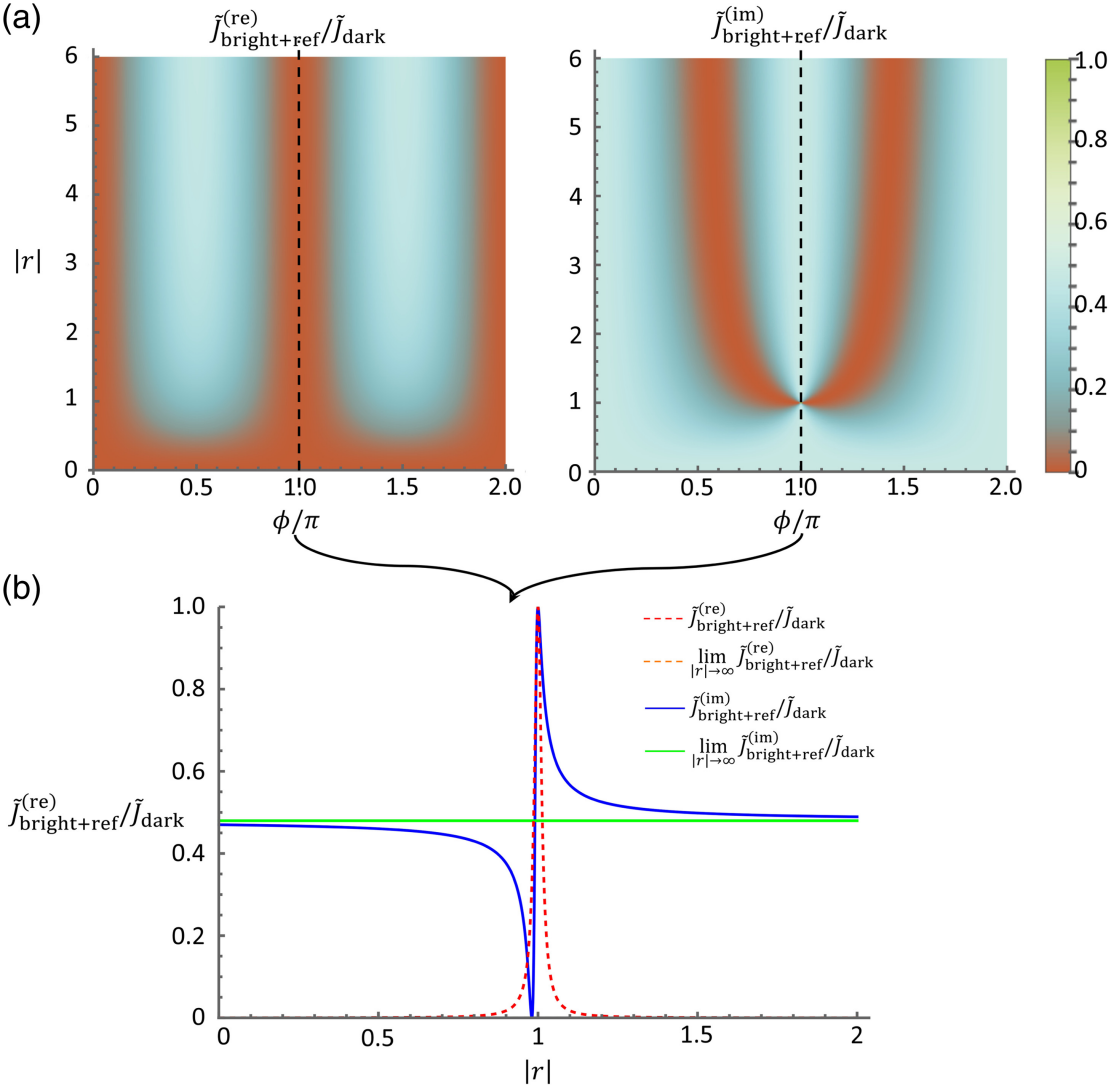
Ratio between the normalized Fisher information in bright-field imaging with reference, ${\tilde{J}}_{\mathrm{bf},\mathrm{int}}$, and in dark-field imaging, ${\tilde{J}}_{\mathrm{df}}$. (a) This ratio is plotted as a function of the relative amplitude, R, and phase, ϕ, of the reference field. (b) This ratio is plotted as a function of the relative amplitude of the reference, R, at a fixed value of the phase to demonstrate its asymptotic behavior compared with the corresponding approximated limit expression.

Unlike conventional direct imaging approaches, the exploration of interferometric detection methods in optical experiments opens new avenues for precision and sensitivity. Subtle variations in the optical path length can be precisely detected, enabling the measurement of quantities such as phase differences with exceptional accuracy. When optical signals are weak, the signal strength can be boosted by interfering with the signal using a strong reference field. The signal boost often allows for the signal to be raised over the level of electronic noise of detectors. However, additional shot noise is introduced into the detection due to the presence of the reference beam. Indeed, in the limit of a strong reference beam, the noise is dominated by the contribution from the reference beam, which allows for shot-noise limited detection of the signal beam. To quantify the potential enhancement in the estimation of the object susceptibility from interferometric detection, we examine the Fisher information in both dark- and bright-field imaging with a coherent reference beam. The ratio of the Fisher information between each detection scheme with reference and the dark field without reference is plotted in Figs. 3(a) and 4(a) accordingly.

The relative phase, ϕ, of the reference beam plays a significant role of determining whether we have constructive or destructive interference with the object field. The interference results in boosted or diminished information with respect to the optical susceptibility perturbation without drastically changing the overall measurement and, consequently, the noise level. Therefore, the Fisher information analyses allow for determining the optimal reference beam intensity and phase relative to the incident.

Given the demonstrated enhancement in sensitivity achieved by suppressing the noise level, it is only natural to question whether dark-field imaging with a reference offers any advantages over its bright-field counterpart. Interestingly, as long as the reference is not fully eliminated, the noise level experiences similar increases in both detection schemes, whereas the information pertaining to the susceptibility perturbation remains constant. This leads to comparable reductions in the Fisher information. However, as the reference strength increases, the interference term gains dominance, leading to the asymptotic behavior of the normalized Fisher information converging to ${\overset{˜}{J}}_{\mathrm{ref}}^{\left(\mathrm{re}\right)}\to H^{2}\;\sin^{2}\;\phi$ for the real-valued susceptibility case and for the imaginary-valued susceptibility case, ${\overset{˜}{J}}_{\mathrm{ref}}^{\left(\mathrm{re}\right)}\to H^{2}\;\cos^{2}\;\phi$. This asymptotic behavior is illustrated by plotting the Fisher information against the relative amplitude and phase of the reference in Figs. 3(b) and 4(b). Notably, the convergence rate is influenced by the object susceptibility or volume, with a slower convergence observed for larger values of these parameters. Nonetheless, the ratio between the Fisher information in either detection scheme with reference and dark-imaging converges rapidly to ∼50% at optimal relative reference phases.

## 5 Discussion

The analysis presented here covers a significant range of parameter space. To make the results more concrete, we shall discuss an illustrative example. We take as an example a molecule-exemplified fluorescent chromophore, with specific numbers for enhanced green fluorescent protein. Although such an example is not definitive, it does establish an estimate for detection that will likely be on the same order of magnitude for the detection of interactions based on electronic resonances. A similar exercise could be implemented for other interactions, but the strategy will be the same. In our example, we choose a wavelength of λ=510  nm and an objective lens with NA=0.95, and we have a beam radius of w=280  nm and an area of $A_{b}=0.122\;\mu m^{2}$. Furthermore, we assume an illumination power of $p_{i}=10\;\mathrm{mW}$, which represents a photon rate of $\phi_{i}=2.6\times 10^{10}\text{ photons}/\mu s$ and a photon flux at the focus of $F_{i}=2.1\times 10^{11}\text{ photons}/\mu s\;\mu m^{2}$.

For enhanced green fluorescent protein (eGFP), we use a ground-state polarizability of $\alpha_{\mathrm{eGFP},g}^{\left(1)\right)}=(1.69+i1.13)\times {\mathrm{nm}}^{3}$ and, in the excited state, a value of $\alpha_{\mathrm{eGFP},e}^{\left(1)\right)}=(-2.48-i1.13)\times {\mathrm{nm}}^{3}$. Clearly, these values will vary over the excitation and emission bandwidth, and these particular values are chosen because they are near the spectral peaks. For a number density of N eGFP molecules, we have a susceptibility of δε=Nα. For our purposes, we assume an eGFP concentration of 1 mM, which leads to $\delta \epsilon_{\mathrm{eff},g}=(1+i0.68)\times 10^{-3}$ and $\delta \epsilon_{\mathrm{eff},e}=(-1.5-i0.68)\times 10^{-3}$.

With a fluorescent molecule, we have multiple modes of detection. Let us first consider fluorescence, for which single-molecule detection is feasible. Here, we assume that the 10-mW incident beam is used for excitation, and accounting for the 60% fluorescent quantum yield, $6.8\times 10^{-8}$ of the incident excitation photons are converted into emitted fluorescent photons. Assuming a collection and detection efficiency of 10%, these fluorescent photons could be detected at a rate of 175  photons/μs. This easily exceeds the threshold for single-molecule level detection. Fluorescent detection benefits from the ability to isolate the signal (fluorescent) photons from the incident photons with spectral filters. Such a luxury is not always available, and the presence of additional background light from the excitation, for example, can add significant noise.

Scattered light detection provides a direct measurement strategy that does not rely on the emission of fluorescent light. As absorption depends on the volume and scattering depends on the square of the volume, the scattered power will remain weaker than the absorbed power until the diameter of the interaction volume exceeds $d\approx 0.53\delta \epsilon_{\mathrm{eff}}^{-1/3}\lambda$. For the value of the eGFP effective susceptibility, an interaction volume diameter of ∼3  μm is required for the level of the scattered power to reach that of the absorbed power. However, the analysis presented in this paper shows that the volume of the interaction does not impact the limiting case of detection, despite the fact that volume plays a key role in the generation of scattered or absorbed light signals. We have seen that the main driver in the normalized Fisher information (i.e., per photon) is the numerical aperture of the imaging and detection optics. To highlight this, consider a sphere with a radius of λ/5 that contains our eGFP sample at 1-mM concentration. In this case, the normalized dark-field Fisher information is ${\tilde{J}}_{\mathrm{df}}=0.299$, which means that the CRLB for detection sensitivity is given by $\delta \epsilon_{\min}=1/\sqrt{N_{i}{\overset{˜}{J}}_{\mathrm{df}}}$. For our scenario, $N_{i}=1.8\times 10^{10}\text{ photons}$, i.e., a 10-mW beam, when we assume a detector with 70% efficiency and a Δt=1  μs integration time. This produces $\delta \epsilon_{\min}=1.36\times 10^{-5}$ for dark-field detection, and this result is independent of the detection concentration.

For bright-field detection, we have ${\tilde{J}}_{\mathrm{bf}}=0.200$ for an imaginary-valued susceptibility, which results in a detection sensitivity of $\delta \epsilon_{\min}=1.67\times 10^{-5}$. This bright-field detection is driven by the imaginary component of the effective susceptibility because the normalized Fisher information from the real-valued component is vanishingly small. For the real-valued case, the normalized Fisher information becomes concentration-dependent, with ${\tilde{J}}_{\mathrm{bf}}=2.33\times 10^{-8}$ and $\delta \epsilon_{\min}=4.89\times 10^{-2}$ for a 1-mM concentration compared with ${\tilde{J}}_{\mathrm{bf}}=2.33\times 10^{-14}$ and $\delta \epsilon_{\min}=48.9$ for a 1-μM concentration. Nearly any other configuration of interferometric detection perform poorly for bright-field detection of a real-valued susceptibility, with the exception of the interferometric null, in which the optimal dark-field detection sensitivity can be recovered. The other interesting cases pointed out earlier, for a strong reference, lead to the bright-field Fisher information, and to the interferometric null, which produces the dark-field case. For a change in volume, the Fisher information values scale with the square of the volume, but the relative strength of the dark to bright field remains the same.

A downside of direct detection, such as optical absorption or scattering, in which we look for a change in the signal power of the optical beam is that the signal can be confounded by other losses that can occur, such as spurious scattering or absorption. Some specificity is necessary to enable sensitive detection without ambiguity from other background contributors. As mentioned above, one method of specific detection is fluorescence, in which the signal can be isolated based on optical filters. Yet, fluorescence detection rates are bound by the spontaneous emission rate. Two label-free methods are also capable of spectroscopic isolation of the molecular signal of interest: TA/SE and photothermal (PT) detection. In both cases, a nonthermal equilibrium is prepared by a pump pulse that modulates the signal extracted by a probe pulse. Because the modulation by the pump pulse is tuned to target a particular spectroscopic transition, specificity can be reliably achieved. In the case of TA/SE, the polarizability of the molecular interaction is generally imaginary, so high Fisher information detection is possible with a bright-field scenario; however, with interferometric detection, the limiting case of dark-field detection is also possible. In the case of PT, the thermally induced susceptibility perturbation is real-valued. As a result, simple bright-field detection will perform poorly, and dark-field scattering or optimized interferometric detection is preferred. The calculations and typical values of normalized Fisher information follow the same approach as with direct absorption and scattering. In the case of PT, there is a further boost to the normalized Fisher information of $B^{\left(\mathrm{PT}\right)}$ over the initial energy deposition step from absorption. Similarly, for TA/SE, there is a boost given by $B^{\left(\mathrm{TA}/\mathrm{SE}\right)}\approx \eta_{\mathrm{pu}}/\eta_{\mathrm{se}}$ that scales as the ratio of the stimulated absorption to the stimulated emission, indicating a more rapid turnover of excitation and emission cycles. In both cases, the boosting factor will increase the effective linear susceptibility perturbation. However, as we have seen, only in the case of a real-valued susceptibility with bright-field detection does the value of the effective susceptibility perturbation play a role, and thus, an enhancement from this amplification factor is only anticipated in the case of the unoptimized scenario of bright-field detection of a PT signal, in which the detection limit will be improved by the enhancement factor. It is worth emphasizing that the key advantage in PT and TS/SE is the ability to better isolate the target signal with pump modulation. The lifetime of signal decay is also a factor that can be used to isolate particular signals.

The other modalities are all nonlinear signals that exhibit enhancements from pulsed excitation and beam intensities. In the case of SHG and THG, these signal generation mechanisms are intrinsically dark-field scattering modes and thus can reach the optimal detectivity, provided that shot-noise limited detection is possible. Although CARS and CSRS are only driven by resonant vibrational scattering there is always a strong interference from nonresonant scattering from electronic resonances, which further complicates their detailed analysis. SRL/SRG are pump–probe methods that are dominated by an imaginary-valued susceptibility on vibrational resonance and thus can be readily optimized through bright-field or interferometric detection. Finally, ISRS is also a pump–probe method that usually produces a purely real susceptibility perturbation. Here, direct bright-field detection, although possible, is disfavored, and interferometric detection^78^ is preferred. Note, however, that there are complexities involved with ISRS due to spectral scattering that induces additional subtleties in the analysis.^42^

## 6 Conclusions

We have presented a comprehensive examination of signal detection methods in label-free imaging, culminating in the development of a universal signal model for measuring the optical susceptibility of sub-wavelength particles applicable across various imaging modalities. By leveraging Fisher information analyses, we have explored the sensitivity of each modality, assuming that cases in optical susceptibility are limited to purely real or imaginary within the shot-noise limit.

In the model, we assume a dipole source that is polarized in the plane parallel to the optical axis and exemplified by the target molecule at some concentration, N, that is contained within a sphere of radius a. Within the sphere, we consider that the set of target molecules present are subject to illumination and that the radiation produced by the optical interaction can be modeled as an effective linear susceptibility, $\delta \epsilon_{\mathrm{eff}}$, that is computed from the time-averaged signal that is collected on a detector. This effective susceptibility is defined such that the average scattered and extinguished power may be computed from the standard linear cross-section expressions.

Moreover, a generalized detection scenario that admits the possibility of including a coherent reference beam is included. The introduction of a coherent reference beam offers a number of strategies for manipulating the information that can be extracted from an optical detection experiment. The classic strategy is to use interference to boost the strength of the detected field, which has an added benefit of linearizing the signal intensity with respect to the signal field. However, the addition of the reference field also increases the level of shot noise in the measurement, which will reduce the Fisher information and lead to a loss of information. However, by controlling the amplitude and phase of the reference field, we can manipulate the Fisher information into favorable scenarios. With a large reference beam, i.e., R→∞, we can mimic bright-field imaging, but now, with the capability of adjusting the relative phase between the scattered field and the reference, we can reach the limit of bright-field detection Fisher information, $H^{2}$. We can also set the interferometer to null the incident field, allowing for mimicking of dark-field detection even if the light is collected in a forward scattered direction as occurs in a standard bright-field measurement. In this scenario, we reach the limit of dark-field detection Fisher information of 4Γ. We find that all other configurations are bounded by these measurement possibilities.

As we attain two distinct normalized Fisher information maxima, the question arises as to which experimental scenario will provide more information, and thus more sensitivity, for the detection of a target molecule. To evaluate this question, we defined the ratio of the dark- to bright-field Fisher information quantities as $\rho_{N}$, which is given in Eq. (13). The values depend only on the numerical aperture, ranging from zero for no NA to just more than 3 for an NA of unity. When $\rho_{N}>1$, dark-field detection is favorable, and this threshold occurs for NA = 0.62. Thus, for microscopy, we are invariably under a scenario dominated by dark-field detection. However, because our model is restricted to a radius of an interaction volume that is small compared with the wavelength, extrapolation to an NA<0.5 is difficult to assess, but our results are relevant for application to high-resolution label-free optical microscopy. The conclusion is that, for reasonable experimental scenarios, the maximum Fisher information is attained in dark-field imaging without reference and equivalently in bright-field imaging only with reference when the reference cancels out the incident field. In cases in which a reference beam is necessary for signal detection facilitation, opting for a relatively large beam intensity is advisable for achieving improved estimation precision. It is important to remember that this analysis relies on the assumption of shot-noise limited detection that, when relative intensity noise from sources and electronic noise dominate over the shot noise, other conclusions could be reached.

In this calculation, we considered the limiting case of ideal optical detection, in which we need to consider only shot noise in the detection. Such a model neglects the effects of noise in the detector, such as Johnson noise and dark current, as well as relative intensity noise (RIN) that is present in optical sources. We make these assumptions because we are primarily interested in the limiting case of weak signal detection and, thus, low dark-field signal flux. At such low signal levels, RIN becomes negligible, and the dominant noise that we must contend with is dark current noise. Dark current is modeled as a Poisson process and thus is an additive noise contribution in the denominator of the Fisher information. The key issue is that the dark counts should be lower than the dark-field signal flux to prevent long signal integration times. Under such conditions, the conclusions that we draw here are widely applicable.

## Data Availability

The mathematic code for the analysis is included as formulae presented herein.
